# Targeting inflammation in cancer therapy: from mechanistic insights to emerging therapeutic approaches

**DOI:** 10.1186/s12967-025-06583-3

**Published:** 2025-05-26

**Authors:** Saad Bakrim, Meriem El Fessikh, Hamza Elhrech, Nasreddine El Omari, Mohammed Amanullah, Long Chiau Ming, Said Moshawih, Abdelhakim Bouyahya

**Affiliations:** 1 Geo-Bio-Environment Engineering and Innovation Laboratory, Molecular Engineering, Biotechnology and Innovation Team, Polydisciplinary Faculty of Taroudant, Ibn Zohr University, Agadir, 80000 Morocco; 2 Laboratory of Human Pathologies Biology, Faculty of Sciences, Mohammed V University in Rabat, Rabat, Morocco; 3 High Institute of Nursing Professions and Health Techniques of Tetouan, Tetouan, Morocco; 4 Department of clinical Biochemistry, College of Medicine, King Khalid University, Abha, Kingdom of Saudi Arabia; 5 Datta Meghe College of Pharmacy, Datta Meghe Institute of Higher Education and Research (deemed to be University), Sawangi (M), Wardha, India; 6 Faculty of Medical and Life Sciences, Sunway University, Sunway City, Malaysia; 7 Department of Pharmaceutical Sciences, Faculty of Pharmacy, Al-Ahliyya Amman University, Amman, Jordan

**Keywords:** Inflammation, Cancer, Innate immunity, Inflammatory mediators, Signaling pathways, Anti-inflammatory drugs, Inflammation-targeted therapy

## Abstract

Inflammation is a complex and finely tuned component of the host defense mechanism, responding sensitively to a range of physical, chemical, and biological stressors. Current research is advancing our grasp of both cellular and molecular mechanisms that initiate and regulate interactions within inflammatory pathways. Substantial evidence now indicates a profound link between inflammation, innate immunity, and cancer. Dysregulation of inflammatory pathways is known to be a pivotal factor in the induction, growth, and metastasis of tumors through multiple mechanistic pathways. Basically, the tumor microenvironment (TME), characterized by dynamic interplay between cancerous cells and surrounding inflammatory and stromal cells, plays a central role in these processes. Increasingly, controlled acute inflammation is being explored as a promising therapeutic tool in certain types of cancer. However, inflammatory cells in the TME exhibit remarkable plasticity, with shifting phenotypic and functional roles that facilitate cancer cell survival, proliferation, and migration, especially under chronic inflammatory conditions. Additionally, signaling molecules associated with the innate immune system, like chemokines, are co-opted by malignant cells to support invasion, migration, and metastasis. These findings underscore the need for deeper insights into the mechanisms connecting inflammation to cancer pathology, which could pave the way for innovative diagnostic approaches and targeted anti-inflammatory therapies to counter tumor development. The current review underlines the critical involvement of inflammation in cancer development, examining the connection between the immune system, key inflammatory mediators, biomarkers, and their associated pathways in cancer. We also discuss the impact of inflammation-targeted therapies on anticancer signaling pathways. Furthermore, we review major anti-inflammatory drugs with potential applications in oncology, assessing how inflammation is modulated in cancer management. Lastly, we outline an overview of ongoing discoveries in the field, highlighting both the challenges and the therapeutic promise of targeting inflammation in cancer therapy.

## Introduction

Inflammation is an early and evolutionarily conserved phenomenon characterized by the stimulation, recruitment, activity, and modulation of innate and adaptive immune cells. While initially identified as a fundamental mechanism in host defense against infections, inflammation is equally essential for promoting functions such as tissue remodeling, renewal, and repair [1]. Often described as “regular hyperplasia inflammation,” this phenomenon is closely tied to the body’s recovery cycle. Tissue damage triggers an intricate network of messenger molecules, which recruit white blood cells—including monocytes, eosinophils, and neutrophils—from the bloodstream to the damaged site, enhancing the repair process [2]. This intricate biological response promotes tissue healing, cell proliferation, and the clearance of debris and defective cells, ultimately maintaining tissue homeostasis [3].

Concern about the link between inflammation and cancer has grown considerably in recent decades. Research into the relationship between inflammation, the immune system, and tumor growth has increased, demonstrating that inflammatory processes play a significant role in the pathophysiology of cancer, extending far beyond tissue healing [4]. Despite progress in cancer management modalities, such as radiotherapy, chemotherapy, and immunotherapy, cancer remains one of the most common causes of death globally. According to recent projections by the World Health Organization (WHO) and the Global Cancer Observatory (GCO), nearly 16.3 million people are expected to die from cancer-related causes by 2040. In addition, 28.4 million cases of cancer are expected worldwide by 2040, corresponding to an overall rise of 47% compared to 2020, with an especially marked progression in transition countries (64–95%) versus developing countries (32–56%), linked to demographic transformations and a worldwide progression of related cancer risk factors [5]. Consequently, contemporary cancer research has progressively shifted from a tumor cell-centric approach to a more holistic vision that recognizes the tumor microenvironment as a crucial factor in determining disease progression and treatment efficiency [6]. The TME, comprising stromal cells like immune cells, fibroblasts, and vascular cells, interacts dynamically with tumor cells. Notably, the inflammatory TME has emerged as a critical determinant impacting tumor development, metastasis, and resistance to chemotherapies [7].

Historically, the hypothesis underlying the relationship between cancer and inflammation has long been recognized. As early as 1863, Rudolf Virchow observed the association of inflammation with tumorigenesis, describing it as a common precursor to cancer development [8]. Later, in the 1970s, Alexander Haddow hypothesized that the “overheating” of wounds could lead to malignancies [9]. Similarly, Harold F. Dvorak has notably characterized tumors as “wounds that do not heal”, emphasizing the corresponding similarities between wound-healing mechanisms and the growth of tumor stroma [10]. By the 1990s, experimental evidence revealed that surgical stress, induced by tissue injury could activate angiogenesis, thereby promoting tumor growth in models such as nude mice [11].

Contemporary research has expanded this fundamental knowledge, demonstrating that cancer initiation and growth can be mitigated by inhibiting inflammatory cells and their mediators, as well as by targeting genomic alterations linked to inflammation [4]. This highlights the potential of inflammation as a therapeutic target in tumor research [12]. Within the TME, diverse inflammatory cells—such as mast cells, eosinophils, neutrophils, tumor-associated macrophages (TAMs), immature myeloid cells, lymphocytes, natural killer (NK) cells, and dendritic cells (DCs)—contribute to cancer development [13]. These cells produce a myriad of pro-inflammatory and cytotoxic mediators, such as free radicals, interleukins (e.g., IL-1, IL-6, IL-17), monocyte chemoattractant protein-1 (MCP-1), tumor necrosis factor (TNF)-α, and transforming growth factor (TGF)-β. Collectively, these molecules promote major processes such as autophagy, angiogenesis, immunosuppression, cancer cell proliferation, metastasis, and resistance to radiotherapy and chemotherapy [14, 15].

A deeper grasp of inflammation mechanisms within the EMT could reveal precise therapeutic solutions offering promising long-term anti-cancer benefits [16]. Clinical studies consistently recommend improving lifestyle, by following a healthy diet, exercising regularly, reducing alcohol consumption, and stopping smoking, which can significantly reduce inflammation and cancer-related mortality [17]. Additionally, emerging findings suggest that common non-steroidal anti-inflammatory drugs (NSAIDs) such as aspirin, and cyclooxygenase-2 (COX-2) blockers, may be effective in preventing cancer, even though outcomes remain somewhat controversial and context-dependent [18].

Although the connection between inflammation, innate immunity, and cancer is now well recognized, many cellular and molecular pathways underlying this relationship remain poorly understood. This review aims to elucidate the multifaceted role of inflammation in tumor development and progression by exploring the interaction between the immune system, key inflammatory mediators, biomarkers, and intracellular pathways. We also investigate how inflammation-related signaling pathways modulate anticancer therapeutic responses. Additionally, we discuss the potential of anti-inflammatory drugs in modern cancer management. Finally, we debate recent advancements and emerging challenges in inflammation-targeted cancer therapy, providing insights into this area of research.

## Overview of inflammation in Cancer treatment

Although inflammation is tightly linked to various types of cancer and acts as a central mediator of tumorigenesis *via* several signaling pathways, some tumors may emerge without significant inflammatory implications, prompted instead by hormonal disruptions, genetic alterations, or other non-inflammatory mediators [19, 20]. Within the TME, cancer-related inflammation is integral to the complex interactions between immune cells and cancer cells, mediated through intricate signaling networks [21]. For instance, glycine transporter 1 (GlyT1), a key regulator of glutamatergic transmission in the spinal cord, has been identified in activating microglia and the complement system. Chemokines such as IL-36, which act *via* the IL-36 receptor (IL-36R) expressed in certain cancer cell lines, are part of the IL-1 cytokine family. These proteins exhibit antimicrobial properties and are implicated in autoimmune disorders, further underscoring the multifaceted involvement of inflammatory pathways in oncogenesis [22–25].

Interestingly, studies on genetically engineered murine cancer cell lines, such as B16F10, demonstrate how oxidative and reductive processes in melanin metabolism can influence the phenotypes of cancer cells [26]. The interplay of oxidative states within melanosomes and the involvement of specific antibodies underscore the complexity of cancer-related inflammation [27].

### Exploring the relevance of inflammation in Cancer formation

The phenomenon of inflammatory oncotaxis, where tumors preferentially develop at sites of chronic inflammation or local tissue injury, is well understood. Interferons, TNFs, and interleukins are examples of pro-inflammatory cytokines that play pivotal roles in initiating and regulating immune responses against cancer cells. These cytokines can activate immune cells, including natural killer (NK) cells and T cells, to detect and remove cancer cells [28, 29]. Conversely, immunosuppressive cytokines like TGF-β and IL-10, frequently produced by tumor cells or TAMs, facilitate immune evasion, enhancing tumorigenic and metastatic abilities [30, 31].

The TME comprises diverse cellular and extracellular ingredients, notably fibroblasts, immune cells, endothelial cells, and extracellular matrix (ECM) molecules. Inflammatory cells like DCs, neutrophils, macrophages, and T cells are attracted to the tumor site and engage in dynamic interactions with cancer cells. These interactions are governed by an equilibrium between pro-inflammatory and anti-inflammatory signals, which can either inhibit or promote tumor progression [32].

Molecular pathways such as those mediated by chemokines are central to inflammatory oncotaxis. Chemokines, like CCL2 (MCP-1), CXCL8 (IL-8), and CXCL12 (SDF-1), are highly expressed in the TME and recruit immune cells to the site of inflammation, supporting tumor formation and metastasis [33]. Additional factors such as prostaglandin E_2_ (PGE_2_), growth hormones, and extracellular vesicles further modulate inflammatory oncotaxis and tumor progression [30]. Pro-inflammatory cytokines like IL-6, IL-1β, and TNF-α, generated in response to tumor antigens, activate NK cells and cytotoxic T cells. These immune cells release cytotoxic molecules namely perforin and granzymes, directly targeting tumor cells [34]. However, tumor cells counteract this by releasing immunosuppressive cytokines and recruiting TAMs, which polarize into an M2-like phenotype under the influence of TME signals such as IL-4, IL-10, andTGF-β [35]. M2 TAMs promote angiogenesis, ECM remodeling, and immunosuppression, contributing to tumor progression and poor prognosis [36].

TAMs exhibit a central action in epithelial-mesenchymal transition (EMT) and constitute up to 50% of cells in solid tumors [37]. They are recruited from circulating monocytes by growth factors and chemokines including CCL2, IL-1β, and vascular endothelial growth factor (VEGF), and subsequently adopt an immunosuppressive phenotype, reinforcing the vicious cycle of tumorigenesis [38].

To develop innovative therapeutic approaches, it is essential to understand the highly interdependent relationship between inflammation and cancer. Targeting pro-inflammatory cytokines or immune checkpoints involved in immune evasion offers promising avenues for cancer therapy. Additionally, modulating the TME to favor anti-tumor immunity and suppress tumor-supportive inflammation can enhance the efficacy of immunotherapies [20, 39–43].

Recent advances in laboratory and clinical investigations have solidified the link between inflammation and cancer progression [41]. Chronic inflammation not only increases tumor survival but also creates an environment conducive to immune suppression and resistance to therapy. Developing anti-inflammatory pharmaceutical interventions (APIs) and immunotherapies targeting the inflammatory response holds significant potential in sensitizing the immune system to remove tumor cells [44] ; [45, 46].

## Inflammatory characteristics and the immune system’s ability to counteract them

Tumors are complex, organ-like structures comprising a diverse array of nonmalignant stromal, inflammatory, and immunological cell types [47], as well as tumor-associated vascular and ECM components [48]. These tumors actively recruit host-derived substances and cells [49], manipulating or suppressing their functions to promote tumorigenesis, immune evasion, invasion, and metastasis. In the early stages of tumor formation, a cascade of events facilitates the survival, proliferation, and genetic alterations of reactive cells. This cascade underlines the fact that inflammation and cancer are closely linked. This complex interaction involves various sophisticated processes that considerably affect tumor development and progression.

Exploring the multifaceted relationship between inflammatory mediators, tumor cells, and their surrounding microenvironment is crucial in oncology. These interactions involve sophisticated signaling pathways that orchestrate immune modulation, angiogenesis, ECM remodeling, and immune escape mechanisms. Advances in deciphering these pathways hold promise for the design of targeted immunotherapeutic interventions and individualized therapies. By identifying inflammatory molecules and pathways that fuel tumor progression, it becomes feasible to design therapies aimed at disrupting these mechanisms, thereby halting cancer development.

Continuous research on the intersection of inflammation and cancer offers the potential for breakthroughs in immunotherapy. These efforts are anticipated to enhance the efficacy of treatments, improve patient outcomes, and redefine therapeutic paradigms in oncology [50, 51].

### Immune system and inflammatory mediators

The immune system, alongside inflammatory mediators, contributes to cancer progression in two ways—either promoting or inhibiting tumorigenesis. Immune cells like mast cells, neutrophils, lymphocytes, myeloid-derived suppressor cells (MDSCs), and TAMs significantly influence the TME [52–54]. These cells contribute to a chronic inflammatory state that supports cancer formation, development, and metastasis.

Chronic inflammation is often accompanied by alterations in the microbiota, collectively termed dysbiosis [55]. Decreased microbial diversity and an excess of pro-inflammatory microbial species are typical hallmarks of dysbiosis, creating a microenvironment conducive to cancer growth [56, 57]. This imbalance in the microbiome has been implicated in various cancers, where microbial-derived metabolites and immune-modulating factors fuel tumor-promoting inflammation [58–60].

Inflammatory pathways are mediated by the recruitment of immune and non-immune cells to sites of infection, injury, or tissue lesion [21]. These recruited cells, including macrophages, DCs, and lymphocytes, release a range of chemokines, growth factors, and cytokines, forming a pro-inflammatory microenvironment [61]. Key signaling molecules, such as pattern recognition receptors (PRRs), activate downstream cascades mediated by signal transducer and activator of transcription (STAT) proteins, perpetuating inflammation [62]. Persistent inflammation fosters the accumulation of reactive nitrogen species (RNS) and reactive oxygen species (ROS), creating oxidative stress that damages DNA, proteins, and lipids [63]. This oxidative stress drives genetic mutations, epigenetic alterations, and immune suppression, which collectively promote tumor progression [64].

Although the immune system can induce antitumor reactions, malignant tumors tend to use inflammatory mechanisms to avoid immune monitoring [65]. Cytokines released during chronic inflammation can shift immune responses toward tumor-promoting phenotypes. Indeed, macrophages polarized to an M2 phenotype under the influence of factors secreted by tumors like IL-4, IL-10, and TGF-β support angiogenesis, ECM remodeling, and immune suppression [66]. Similarly, MDSCs suppress T cell induction, inhibit NK cell cytotoxicity, and enhance regulatory T cell (Treg) activity, further weakening the anti-tumor immune response [67, 68].

This intricate interplay reveals the paradoxical relevance of inflammation in cancer. While acute inflammation may initially limit tumor growth by recruiting cytotoxic immune cells, chronic and unresolved inflammation ultimately fosters an environment that favors tumor survival and immune escape [69]. Targeting these inflammatory mediators and their associated pathways is a promising approach to restore immune competence and inhibit tumor progression. Research into the modulation of inflammatory pathways and the reprogramming of the TME continues to provide valuable knowledge for the development of innovative cancer therapies.

### Immunoregulation and stromal mediators in tumor inflammation

Treg cells are essential for preserving immune suppression, which is always repressed in a healthy immunological environment in order to preserve the stability of the organism. Nevertheless, it is more likely that inhibition of the immunological capabilities of Tregs during carcinogenesis promotes tumor cell migration and proliferation, ultimately leading to tumor progression [70]. On the other hand, Tregs are actively regulated in areas of inflammation, as demonstrated by Lazarski et al. (2008). Inflammatory cytokines, such as TNF-α and IL-6, have been shown to reduce FOXP3 expression, thereby compromising the stability and suppressive activity of Tregs. This implies that Treg plasticity can be modulated by the local inflammatory milieu, affecting immune regulation at inflammatory sites and eventually transforming them into effector-like cells [71]. According to Clarke et al. (2006), colorectal cancer patients exhibit a markedly higher frequency of CD4⁺ CD25⁺ FOXP3⁺ regulatory T cells in both tumor tissue and peripheral blood compared to healthy individuals. These Tregs have been demonstrated to be functionally suppressive, meaning they can prevent effector T cells, particularly those involved in anti-tumor immunity, from proliferating and producing cytokines [72]. In solid tumors, Tregs play a crucial role in preventing the immune response and promoting immunological escape. Several mechanisms are involved. Indeed, there are cytokine-mediated signaling pathways. For example, a previous study by Ahmed et al. [54] demonstrated that Tregs predominantly originated from a subset of CD4 + T cells and that they primarily express CD25. Upon IL-2 binding to the high-affinity IL-2 receptor, CD4 + CD25 + Tregs competitively inhibit the immune response to tumor cells. IL-2 is one of the most potent cytokines stimulating effector T cells generation [73, 74]. Perforin and granzyme B, both of which destroy effector T cells and thereby contribute to the immune suppression of tumors, are also expressed by CD25 + Tregs in peripheral blood [75, 76].

In addition, maturation of tumor surface antigens is affected by Treg expression of cytotoxic T cell antigen 4 (CTLA-4), and immune evasion of tumor cells results from the inability of anti-CTLA-induced immature T cells to recognize tumor surface antigens. In melanoma models, tumor prognosis was somewhat improved by enhanced immune activity following anti-CTLA-4 therapy [70]. Furthermore, Tregs expressing CTLA-4 stimulate the release of indoleamine 2,3-dioxygenase (IDO) by interacting with CD80/86 on the surface of antigen-presenting cells (APCs). An enzyme called IDO is required for tryptophan metabolism. T-cell dysfunction caused by increased tryptophan in TME inhibits the protective immunological response of T-cells against malignant tumors [77–79]. Simultaneously, stromal fibroblasts, particularly cancer-associated fibroblasts (CAFs), are often exposed to persistent NF-κB activation in the TME. CXCL12, IL-6, IL-8, and MMP are among the cytokines and chemokines released by these fibroblasts in response to NF-κB activation. In addition to remodeling the TME, which facilitates the invasion and spread of tumor cells, these factors attract and activate T lymphocytes and other immune cells. In addition, fibroblasts participate in complex signaling relationships with immunological and tumor cells via the NF-κB cytokine network, which ultimately controls local inflammation and induces immune evasion [80, 81]. In addition, CAFs maintain chronic, unresolved inflammation within the TME and also promote the recruitment of Tregs [82]. Recently, Deng, Y et al. showed that CAFs play an essential role in the formation of the TME in colitis-related colorectal cancer. By stimulating angiogenesis, promoting EMT, and maintaining local immunosuppression, they actively promote tumor growth. Through their close interactions with intestinal macrophages and other immune cells, CAFs produce growth factors (TGF-β) and chemokines (CXCL12) that influence macrophage polarization towards the M2 phenotype, resulting in an effective tumor-promoting and anti-inflammatory environment [83]. Thus, Zhou et al. (2025) in their recent study highlight the need to focus on the diversity of CAF subtypes and their dynamic interactions *via* signaling pathways with immunological and tumor cells, which could provide new therapeutic approaches for cancer treatment [84].

Together, these components form a complex system of stromal signaling and immune regulation that drives tumor-promoting inflammation and hinders the effectiveness of antitumor immunity.

## Signaling pathways associated with inflammation in Cancer

Understanding the intricate mechanisms by which inflammation promotes carcinogenesis and how tumor cells subvert anti-cancer immune responses is a primordial field of cancer investigations. Identifying the key signaling pathways implicated in cancer-induced inflammation could point to innovative approaches in early detection, prevention, and therapeutic interventions. Among these, the transcription factors STAT3 and nuclear factor kappa-light-chain-enhancer of activated B cells (NF-κB) are pivotal in associating inflammation with cancer [85]. NF-κB, recognized for its ability to induce survival, immune regulation, and inflammation, has been extensively investigated in various carcinogenic experiments both in vitro and in vivo [86, 87]. Similarly, STAT3 has been identified as an essential governor of inflammation and carcinogenesis, often playing a synergistic role with NF-κB in the establishment of malignancies [88–92]. The dynamic interplay between these pathways is a primary determinant of tumor progression and immune evasion, as will be elaborated in subsequent sections.

### NF-κB signaling pathway

The NF-κB family represents a central hub in inflammatory signaling, innate immunity, and the development of malignant neoplasms [93, 94]. The NF-κB family consists of five protein subunits—RelA (p65), RelB, c-Rel, p50/p105 (NF-κB1), and p52/p100 (NF-κB2)—which can form various homo- and heterodimers [95]. These subunits have a common Rel homology domain (RHD) that enables DNA binding, dimerization, and interplay with inhibitors of κB (IκBs) [96]. The classification of NF-κB subunits into Class I precursors (p105, p100) and Class II Rel proteins (RelA, RelB, c-Rel) underscores their functional diversity, as Class I precursors undergo proteolytic processing to form active p50 and p52 subunits [97].

**Fig. 1:**
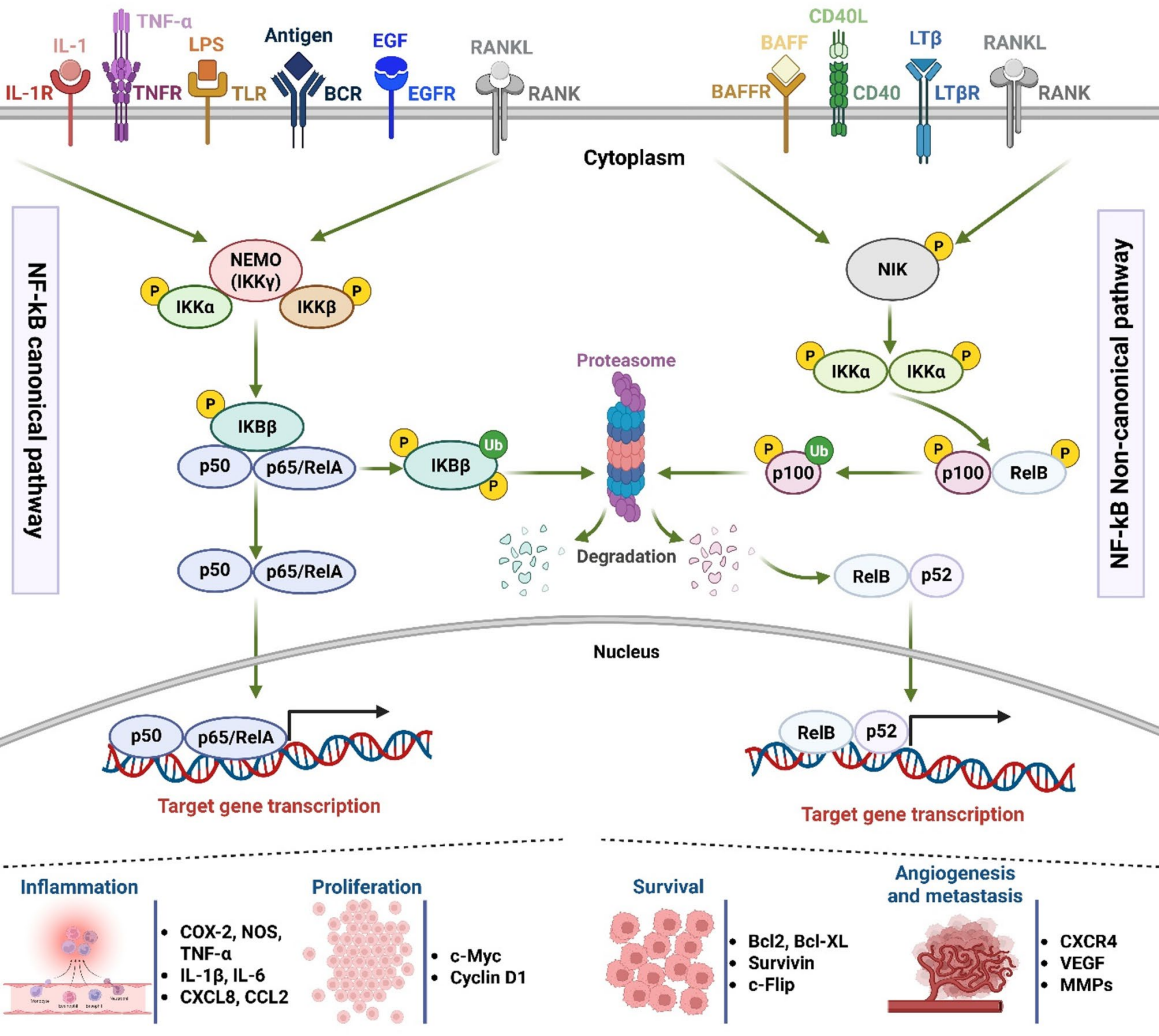
Schematic representation of the inflammatory signaling mechanisms underlying cancer development mediated by the NF-kB signaling pathway. The canonical pathway involves the activation of signaling pathways by pro-inflammatory cytokine receptors (TNFR, IL-1R, TLR), antigen receptors (BCR), and growth factors (EGFR family). These receptors have the ability to trigger the IKK complex, composed of IKKα, IKKβ, and IKKγ (NF-κB, a master regulator (NEMO)). This complex phosphorylates and promotes ubiquitination of IκB, leading to its destruction by the proteasome. After translocation into the nucleus, p65 (RelA)/p50 dimers initiate target gene expression. The cytosolic RelB/p100 complex is inactive, but there is no IκB in the non-canonical pathway. Signals from CD40L, LTβR, and BAFFR trigger activation of NIK. These signals phosphorylate the IKKα/IKKα homodimer, subsequently translocating the phosphate group to p100 C-terminal residues, where it is ubiquitinated and proteasome-transformed to p52. Translocation of RelB/p52 to the nuclear level and increased expression of the target gene constitute the primary endpoints. Both the canonical and non-canonical NF-kB pathways have been identified as intracellular signaling pathways responsible for inflammation-mediated tumors. Abbreviations: BAFF, B-cell activating factor; BCL-2, B-cell lymphoma 2; BCL-XL, B-cell lymphoma-extra-large; BCR, B-cell receptor; CD40, cluster of differentiation 40; COX-2, cyclooxygenase-2; EGFR, epidermal growth factor receptor; IKK, IκB kinase; IL-1, interleukin-1; IL-6, interleukin-6; IL-8, interleukin-8; IκB, inhibitor of NF-κB; LPS, lipopolysaccharide; LTβR, lymphotoxin beta receptor; MMP, Matrix metalloproteinase; NIK, NF-κB-inducing kinase; NOS, nitric oxide synthase; P, phosphate group; RANKL, receptor activator of nuclear factor κ-B ligand; TLR, Toll-like receptor TNF, tumor necrosis factor; TRAF, TNF receptor-associated factor; Ub, ubiquitin moieties

The NF-κB signaling pathway is mediated through two major routes: the canonical and non-canonical pathways [98]. The canonical pathway, triggered by pro-inflammatory stimuli like cytokines (e.g., IL-1β and TNF-α), microbial components, and genotoxic stress, induces transient transcriptional activation of genes implicated in inflammation and immunity [99]. In contrast, the non-canonical pathway, primarily induced by certain members of the TNF receptor superfamily, regulates processes such as lymphoid organogenesis and adaptive immunity [100] (Fig. 1).

#### Mechanisms of NF-κB activation

NF-κB activation implies cleavage of IκBs, which sequester NF-κB dimers in the cytoplasm [101]. Upon stimulation, the IκB kinase (IKK) complex phosphorylates IκBs, marking them for ubiquitination and proteasomal degradation [102]. This allows NF-κB dimers, such as p65 and p50, to translocate into the nucleus [103], where they bind to κB sites on DNA to regulate target gene expression [104]. Intriguingly, molecules like cyclopentenone prostaglandins (e.g., PGE_2_) can modulate this pathway by inhibiting the nuclear translocation of NF-κB, thereby dampening inflammatory responses [105].

#### Activators of NF-κB pathway

NF-κB activation is triggered by diverse stimuli, such as pro-inflammatory mediators (TNF-α, IL-1β, IL-6, and IL-12), chemokines, microbial products, UV radiation, and DNA-damaging agents [106]. These signals converge on the IKK complex, often mediated by adaptors such as TRAF proteins, leading to downstream transcriptional activation of genes encoding chemokines, cytokines, and adhesion molecules that exacerbate inflammation [107–109] ; [110, 111].

#### NF-κB and the innate immune system

Innate immune cells, like neutrophils, DCs, and macrophages, are responsible for generating inflammatory mediators and regulatory substances to eliminate infectious agents. Simultaneously, these cells must activate negative feedback mechanisms to prevent inflammatory reactions [112].

Pathogen-associated molecular patterns (PAMPs), derived from microbial components, and damage-associated molecular patterns (DAMPs), originating from necrotic cells or injured tissue, are detected by PRRs expressed on innate immune cells [113]. Upon activation, PRRs trigger the release of pro-inflammatory mediators, including TNF-α, IL-1β, IL-2, IL-6, IL-8, IL-12, and IFN-γ, alongside chemokines and antimicrobial proteins. These molecules initiate inflammation to eradicate pathogens and repair tissue damage [114].

PRRs are categorized into six groups based on their protein domains and discovery timeline: Toll-like receptors (TLRs) and non-TLRs, including nucleotide-binding and oligomerization domain (NOD)-like receptors (NLRs), retinoic acid-inducible gene I (RIG-I)-like receptors (RLRs), C-type lectin receptors (CLRs), absent in melanoma 2 (AIM2)-like receptors (ALRs), and cyclic GMP-AMP (cGAMP) synthase (cGAS) [115]. These PRRs induce the activation of the classical NF-κB pathway, enabling the release of pro-inflammatory mediators, chemokines, and other inflammatory molecules. Additionally, NF-κB is integral to processes such as granulocyte-macrophage colony-stimulating factor (GM-CSF)-mediated signal transduction and the differentiation of myeloid progenitor cells [116]. Various NF-κB family transcription factors further control the differentiation and development of myeloid-derived cells [117, 118].

#### NF-κB and the adaptative immune system

The adaptive immune system also contributes to inflammation, with the NF-κB pathway having an essential function in modulating and promoting this immune response. When activated, B cells and T cells proliferate and differentiate into effector cells, mediating diverse immunological responses such as cytokine release, cytotoxic T lymphocyte activity, and immunoglobulin secretion by B cells [100].

DCs, as professional antigen-presenting cells, play a pivotal role in bridging innate and adaptive immunity by presenting antigens to CD4 + and CD8 + T cells while secreting cytokines that influence the differentiation of CD4 + T cells into subsets such as Th1, Th2, Th9, Th17, Th22, T follicular helper (Tfh) cells, and Tregs [119]. In fact, the NF-κB signaling pathway is critical for the polarization of specific subsets, notably Th1, Th2, Th17, and Th9 [119–122]. Th1 cells generate IFN-γ and IL-12 to activate macrophages and regulate immune responses against intracellular pathogens [123]. Th2 cells produce IL-4, IL-5, and IL-13, driving responses by basophils, eosinophils, and mast cells, with NF-κB and IL-4 synergistically activating STAT-6 to mediate Th2 cell differentiation [124]; [125]. In addition, the release of IL-17 and IL-22 by Th17 has a crucial effect in attracting monocytes and neutrophils around inflammatory sites, thus enhancing protective immune responses towards pathogenic agents or self-antigens [126]. Subsequent studies have further elucidated that NF-κB regulates Th17 cell differentiation [127]. Notably, Th17 differentiation is significantly influenced by decreased IL-1α, IL-1β, and IL-6 generation by DCs due to RelA deficiency [119]. Concerning T cell receptor (TCR) activation, c-Rel is involved in the generation of IL-21 by CD4 + T cells, an essential cytokine for the development of both Th17 and Tfh cells [128]. Experiments with c-Rel-deficient animals have confirmed altered differentiation of Th17 and Tfh cells [129, 130]. Moreover, NF-κB also facilitates Treg production, with c-Rel being essential for the expression of forkhead box P3 (Foxp3) and Treg formation in the thymus [131]. Additionally, canonical NF-κB signaling-activated RelA and c-Rel influence medullary thymic epithelial cell (mTEC) differentiation by regulating RelB transcription, negative selection of autoreactive T cells, and the growth of Treg cells [132]. Moreover, CD4 + T cells can become increasingly resistant to Treg-induced inhibition of inflammation when p105 is depleted [133].

On the other hand, the proliferation, survival, and functionality of B cells are heavily dependent on the NF-κB pathway [134]. Immature B cells rely on NF-κB signaling downstream of B cell receptors (BCRs), B cell-activating factor (BAFF), and proliferation-inducing ligand (APRIL) receptors. The canonical NF-κB pathway is induced by the pre-BCR and BCR and has an important effect in regulating central tolerance, survival, and differentiation throughout B cell maturation [135]. Besides, suppression of NF-κB c-Rel or RelA subunits, both triggered by canonical NF-κB signals, is critical in regulating high-affinity B cell production in the germinal center response. Notably, RelA is indispensable for the development of plasma cells derived from germinal centers by preventing the upregulation of B-lymphocyte-induced maturation protein-1 (BLIMP1), although it is not important for maintaining the germinal center itself [136].

#### NF-κB signaling and inflammation in Cancer

As a central modulator of inflammatory processes, NF-κB has a central action on tumor pathophysiology and progression. Extensive research has demonstrated its involvement in the onset and development of different malignancies, including gastric cancer, breast cancer, lymphoma, and leukemia [117]. NF-κB activation triggers inflammatory pathways, establishing a positive feedback loop with a rise in chemokine expression (e.g., CXCL8 and CCL2), nitric oxide synthase (NOS), COX-2, and inflammatory mediators (IL-6, IL-8, and TNF-α), thereby fostering tumor development [137].

NF-κB also modulates the expression of multiple anti-apoptotic genes, namely *BIRC5* (Survivin), *c-FLIP*, *B-cell lymphoma-extra large* (*BCL-XL*), and *B-cell lymphoma-2* (*BCL-2*), enabling cancer cell survival ) [138–140]. Moreover, it regulates gene expression linked to tumor invasiveness, metastasis, and angiogenesis, such as C-X-C motif chemokine receptor 4 (CXCR4), VEGF, and MMPs [141]. NF-κB controls the production of mitogenic proteins such as Cyclin D1 and c-Myc, which are essential for cell proliferation [87]. This prolonged activation of NF-κB can therefore lead to resistance against chemotherapy and radiotherapy through its dual actions of inhibiting apoptosis and stimulating cell growth [142].

The interplay between NF-κB, inflammation, and cancer was first highlighted in hepatitis-related liver cancer and colitis-related colon cancer, where NF-κB acts as a key mediator linking chronic inflammation to tumorigenesis [143]. In fact, NF-κB in myeloid cells, particularly lamina propria macrophages, transcribes genes encoding growth factors, enhancing the proliferation of precancerous intestinal epithelial cells [144]. Colitis-driven colon cancer is largely fueled by inflammatory cytokines (e.g., IL-6, IL-23, and TNF), secreted by DCs, macrophages, and TAMs in the lamina propria [145].

#### NF-κB and Cancer stem cell dynamics

NF-κB also promotes cancer cell survival under hypoxic conditions by stimulating hypoxia-inducible factor 1α (HIF-1α) and inducing anti-apoptotic genes such as *Bcl-2*, *Bcl-xL*, and *inhibitors of apoptosis (IAP)* [146]. In ovarian cancer, NF-κB, mediated by HIF-1α, induces cancer stem cell characteristics by increasing sirtuin 1 (SIRT1). Concurrently, HIF-2α activates Wnt/β-catenin and NOTCH signaling, preserving cancer stem cell hallmarks and fostering tumor proliferation [147]. Targeting NF-κB-related pathways, including those mediated by HIF-1α and HIF-2α, seems to be a promising approach for reducing cancer stemness and improving therapeutic outcomes.

#### NF-κB and apoptosis resistance

The suppression or deletion of NF-κB-related genes has been demonstrated to induce cell death. Indeed, knockout (KO) of the *RelA* gene leads to embryonic hepatocyte and fibroblast death in response to TNF-α stimulation, whereas inactivation of the v-Rel oncogene triggers the death of transformed lymphocytes [148]. Beyond apoptosis resistance, NF-κB stimulates cell proliferation by upregulating cyclins such as *Cyclin D1*, *Cyclin D2*, and *Cyclin D3* [149]. Additionally, NF-κB influences p53 stability and cell cycle progression through the NF-κB-dependent activation of the mouse double minute 2 homolog (MDM2), underscoring its multifaceted role in tumor promotion [150].

NF-κB also enhances inflammatory cytokine production in immune cells like macrophages and neutrophils, generating IL-1β, IL-6, and TNF-α. These cytokines, in turn, stimulate the proliferation of malignant and tumor-associated stromal cells, amplifying the TME [151].

#### Implications of NF-κB in Cancer therapy

NF-κB’s transcriptional activity, essential for inhibiting cell death, is frequently upregulated in the TME across various human cancers. Its roles extend beyond inflammation regulation, encompassing tumor invasion, metastasis, and angiogenesis. These observations highlight NF-κB as a pivotal relationship between inflammation and tumor progression.

Targeting NF-κB signaling presents a promising therapeutic strategy. Suppressing NF-κB activity in primary tumor cells can disrupt inflammatory feedback loops and impair mechanisms that enable tumors to evade immune surveillance. However, given the complexity and multifactorial nature of cancer, effective strategies should focus on simultaneously modulating multiple components of the NF-κB pathway [152–154]. Such approaches may offer more comprehensive control of tumor growth and progression while mitigating treatment resistance.

### STAT3 signaling pathway

#### Structural components and isoforms of STAT3

The structural framework of STAT3 comprises several key domains: the transactivation domain (TAD), binding domain, DNA-binding domain (DBD), coiled-coil domain (CCD), N-terminal domain (NTD), and Src homology 2 (SH2) domain. Critical to its activation are the tyrosine 705 (Y705) and serine 727 (S727) residues within the C-terminal domain, which are pivotal for its functional versatility [155]. STAT3 exists in six isoforms: STAT3α, STAT3β, STAT3γ, STAT3δ, STAT3ε, and STAT3ζ, with these variants dictating its diverse biological roles. Among them, STAT3α, the longest isoform, predominantly drives canonical STAT3 signaling [88]. In contrast, STAT3β, lacking the TAD, functions as a tumor suppressor and is related to improved prognoses in cancer patients [156]. The isoforms STAT3γ and STAT3δ arise from proteolytic cleavage during distinct stages of granulocyte and neutrophil maturation, adding another layer of regulatory complexity [157].

#### Canonical and Non-Canonical STAT3 signaling pathways

Canonical STAT3 activation primarily involves phosphorylation at Y705. Cytokines such as IL-6 and various growth factors stimulate receptor-associated tyrosine kinases, activating STAT3 [158]. Phosphorylated STAT3 monomers dimerize through SH2 domain interactions, translocate to the nucleus, and modulate genes related to inflammation, TME, cell proliferation, metastasis, angiogenesis, apoptosis inhibition, and immune evasion [88].

**Fig. 2:**
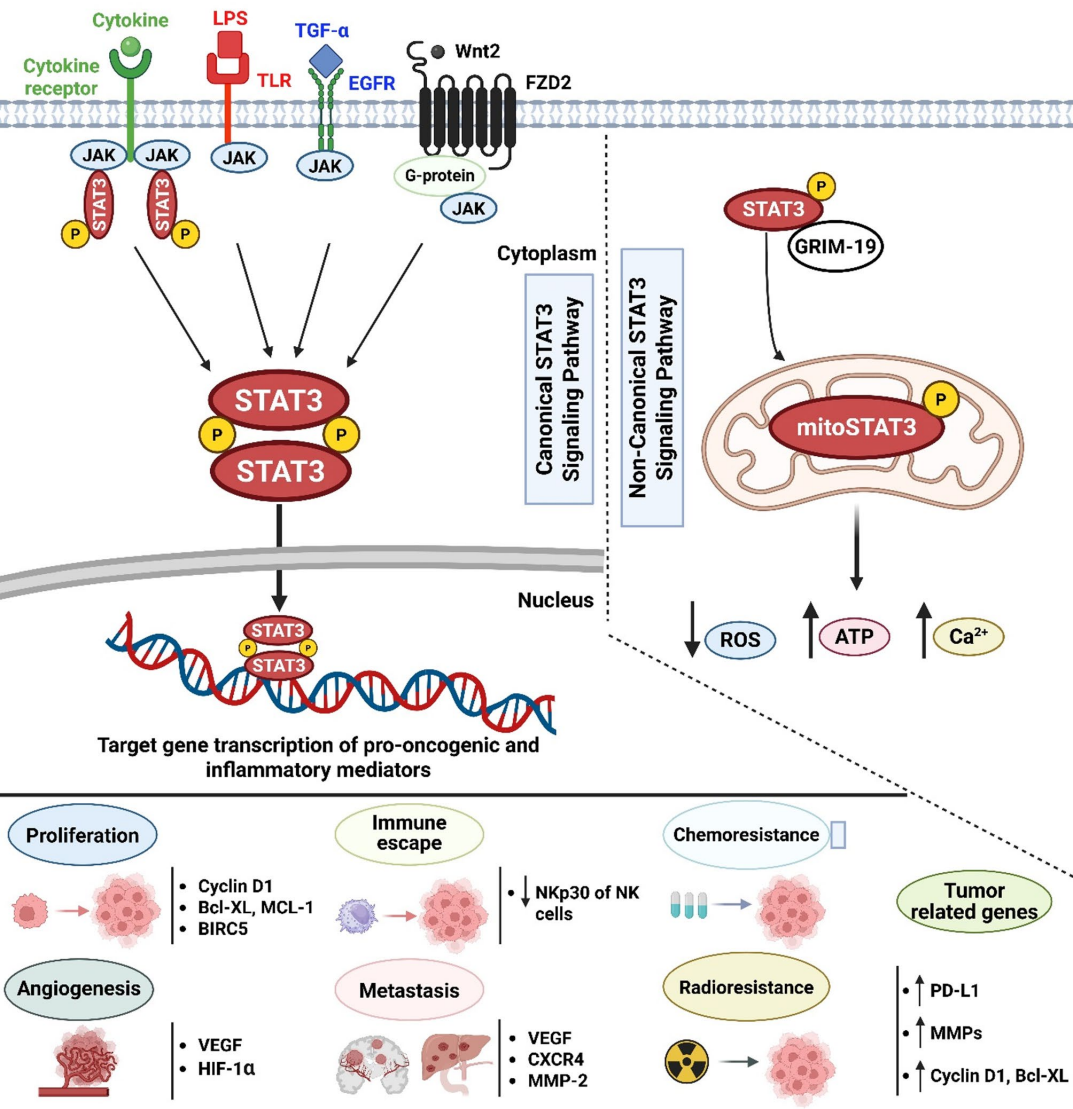
Graphical depiction of mechanisms responsible for inflammatory response mediating the onset of cancer *via* STAT3 signaling pathway. A large number of conserved upstream receptors, known to be important in cancer progression, can activate the canonical STAT3 signaling pathway. Cognate ligands that stimulate these receptors include hormones, cytokines, growth factors, LPS, and others. Following phosphorylation, homodimerisation, and nuclear translocation of the STAT3 dimer, the cytoplasmic STAT3 molecule is activated. The transcriptional machinery responsible for multiple features of cancer progression, including stimulation of immune escape, induction of proliferation, release of pro-inflammatory cytokines, inhibition of apoptosis, angiogenesis, metastasis, chemoresistance, and radio-resistance, as well as an increase in the expression of tumor-related genes, is activated by the STAT3 dimer within the nucleus. GRIM-19 facilitates the recruitment of non-canonical STAT3 signaling to mitochondria. MitoStat3, also known as mitochondrial STAT3, controls events other than transcription, such as ATP generation, reduction of ROS release, and elevation of mitochondrial Ca^2+^. Abbreviations: CXCR4, CXC chemokine receptor 4; STAT3, signal transducer and activator of transcription 3; VEGF, vascular endothelial factor; CXCR4, CXC chemokine receptor 4; NK, natural killer; BIRC5, baculoviral inhibitor of apoptosis repeat-containing 5; NKp30, natural cytotoxicity receptor 1 (NCR1); MMP, Matrix metalloproteinase; LPS, lipopolysaccharide; EGFR, epidermal growth factor receptor; ROS, reactive oxygen species; TNF-α, tumor necrosis factor-α; Ca2+, calcium; PD-L1, programmed death-ligand, BCL-XL, B-cell lymphoma-extra-large; TLR, Toll-like receptor; P, phosphate group; MCL1, myeloid cell leukemia-1; MCL1, myeloid cell leukemia-1; HIF-1α, hypoxia-inducible factor 1α; mSTAT3, mitochondrial signal transducer and activator of transcription 3

Non-canonical STAT3 signaling, while less understood, has received particular attention because of its involvement in cancer. Mitochondrial STAT3 (mtSTAT3) localizes to the electron transport chain (ETC), influencing cellular metabolism, mitochondrial respiration, ROS production, and tumorigenesis. These mitochondrial functions are contingent upon phosphorylation at S727 [159, 160] (Fig. 2).

#### STAT3, inflammation, and Cancer progression

STAT3 performs a dual function in inflammation and immunity within the TME, modulating cytokine-driven responses to either suppress or promote malignancy [161]. While reducing anti-cancer immunity, STAT3 and, to a lesser degree, STAT5 promotes cancer cell growth, survival, and invasion. Persistent STAT3 activation contributes to oncogenic inflammation by counteracting anti-cancer Th1 immune responses induced by STAT1 and NF-κB [162]. Central to this process is the IL-6-GP130-JAK (Janus Kinase) pathway, a significant pro-oncogenic inflammatory axis involving STAT3 [163].

STAT3 is, therefore, an attractive pathway for diverting inflammation in cancer treatments [164]. Cytokine-activated STAT3 homodimers and heterodimeric complexes, in combination with other transcription factors, govern the expression of target genes that oversee cell proliferation, inhibit programmed cell death, stimulate the growth of new blood vessels, facilitate immune evasion by cancers, and foster inflammation that supports tumor development. The complex interaction between several signaling pathways regulating gene transcription underscores STAT3’s crucial role in diverse signaling processes. Chronic inflammation prompts the removal of STAT3 in liver cells, selectively activating STAT3 as needed. For instance, the relationship between cancer development and inflammation was studied using a hepatocellular carcinoma model, examining the occurrence of Treg cells in the TME and STAT3 activation [165–167].

STAT3 is a pivotal transcription factor implicated in tumorigenesis associated with inflammation, particularly contributing to cellular transformation and the growth and survival of inflammation-driven tumors [168]. For example, pro-inflammatory mediators like IL-6 activate the JAK-STAT3 pathway [169]. The IL-6/STAT3 signaling pathway has a key effect on breast cancer, enhancing cell proliferation, survival, and epithelial-mesenchymal transition, notably in triple-negative breast cancer (TNBC) [170]. In solid tumors with elevated proliferating cell nuclear antigen (PCNA) levels and reduced phosphatase and tensin homolog deleted on chromosome ten (PTEN), androgen receptor, caspase-3, and Bcl-2-associated X protein (Bax) levels, high IL-6 amounts contribute to tumor development. Similarly, high STAT3, JAK, Bcl-2, and PCNA expression in musculoskeletal tumors intensifies JAK/STAT3 signaling activation, accelerating tumor growth, inhibiting apoptosis, and promoting angiogenesis [171–173]. Esmaeili et al. [174] recently demonstrated that chronic inflammation-driven colorectal cancer development is facilitated by gut cytokines, the Jak-STAT3 signaling pathway, and N6-methyladenosine RNA. Furthermore, abnormal STAT3 activation is a major driver of pancreatic ductal adenocarcinoma progression through growth factor and inflammatory cytokine activity [175].

STAT3 also regulates macrophage inflammatory responses and other immunological cells [176]. Research shows lactate secreted by tumors promotes breast cancer growth *via* M2 macrophage polarization through extracellular signal-regulated kinases (ERK)/STAT3 pathway induction [177]. Similarly, IL-6/STAT3 pathway activation drives M1/M2 macrophage polarization, accelerating hepatocellular carcinoma progression [178]. Using an ApoE^−/−^ mouse model, metformin was shown to inhibit monocyte differentiation into macrophages *via* AMP-activated protein kinase (AMPK)-mediated STAT3 dephosphorylation [179]. These findings highlight macrophage M1/M2 polarization’s association with the anti-inflammatory and anticancer potential of STAT3 signaling suppressors.

Inflammatory cytokines are key mediators of immune escape in cancer. IL-6 induces anti-apoptotic Bcl-xL protein expression in highly metastatic prostate cancer cells *via* STAT3 activation. Persistent pro-inflammatory signals in the TME create and maintain an immunosuppressive environment, allowing tumors to evade immune detection.

### Collaborative implication of NF-κB and STAT3 signaling pathways

**Fig. 3:**
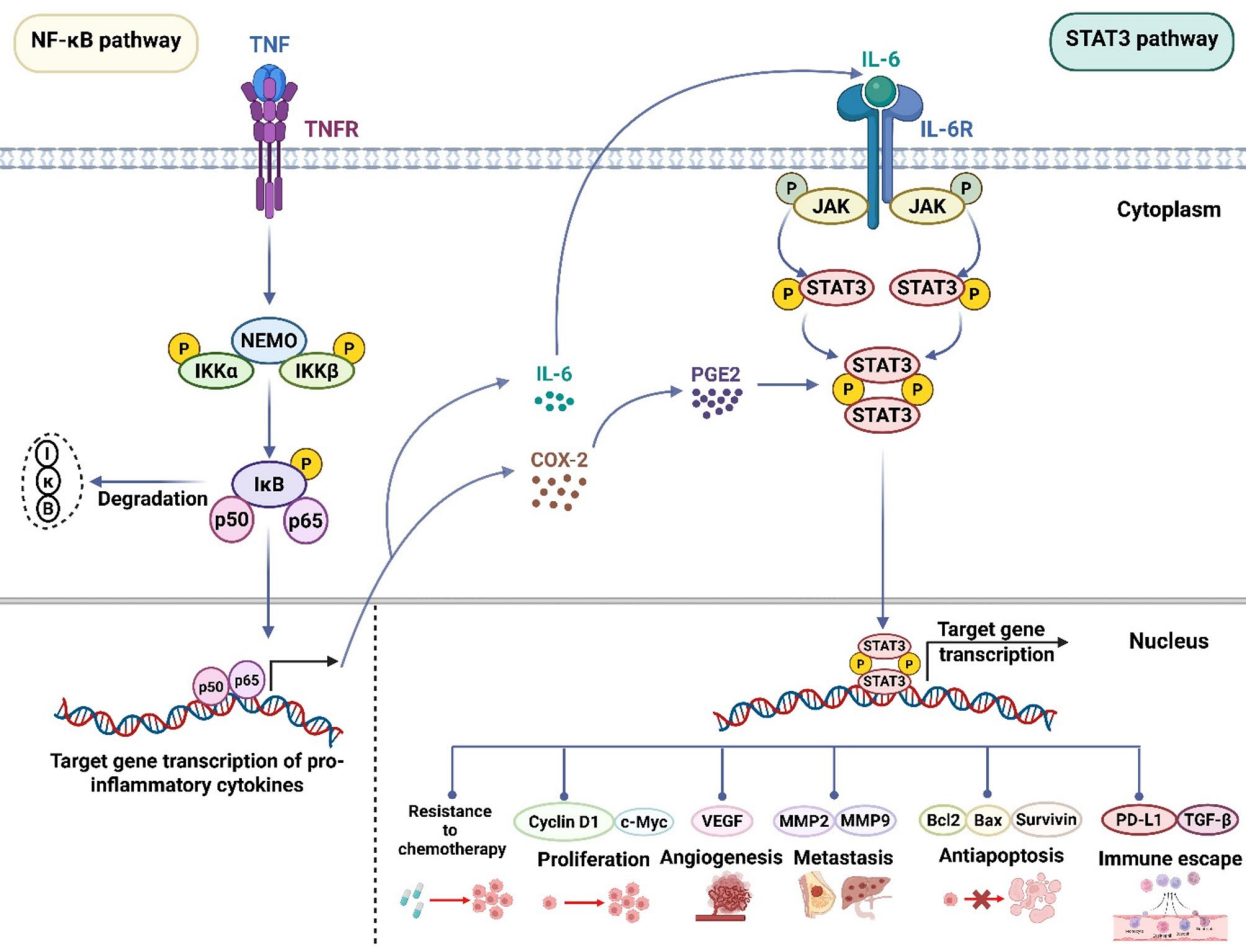
Schematic description of the signaling process demonstrating the intricate cross-talk between NF-κB and STAT3 in inflammation-induced cancer. Both NF-κB and STAT3 have the ability to activate autocrinally and/or paracrinally and to engage in collaborative activation. The NF-κB pathway is triggered by TNF-α, as evidenced by transcription of NF-κB genes, such as IL-6 and COX-2, and phosphorylation and nuclear translocation of NF-κB p50/p65. IL-6 is freshly synthesized and released from the cells, binding to the IL-6 receptor via autocrine or paracrine pathways. As a result, intracellular JAK1/2 kinases and the IL-6R/gp130 complex are activated. In addition, PGE_2_ is generated in response to freshly produced COX-2, causing phosphorylation and activation of STAT3. When JAK1/2 phosphorylates STAT3 proteins, they dimerize, reach the nucleus, and trigger STAT3 transcription, which increases the expression of genes that trigger cancer progression through the various stages of the disease. Abbreviations: BCL-2, B-cell lymphoma 2; BCL-XL, B-cell lymphoma-extra-large; COX-2, cyclooxygenase-2; IKK, IκB kinase; IL-1, interleukin-1; IL-6, interleukin-6; IκB, inhibitor of NF-κB; STAT3, signal transducer and activator of transcription 3; VEGF, vascular endothelial factor; MMP, Matrix metalloproteinase; EGFR, epidermal growth factor receptor; TNF, tumor necrosis factor; TNFR, tumor necrosis factor receptor; PD-L1, programmed death-ligand; PGE_2_, prostaglandin 2; TGF-β, transforming growth factor-β

The intricate interplay between NF-κB and STAT family signaling, especially STAT3, reflects their shared functions in inflammation and cancer [142] (Fig. 3). STAT3 and NF-κB share notable similarities and differences as nuclear transcription factors controlling genes implicated in tumor growth, survival, invasion, angiogenesis, metastasis, and inflammatory cytokines driving cancer [180]. In fact, during activation, the expression of genes associated with immune response, proliferation, and anti-apoptotic processes is controlled by both NF-κB and STAT3. Specific gene networks require the collaborative interaction of these two transcription factors to function effectively. Notably, when inflammatory and immune cells infiltrate tumors, the communication between tumor cells and their microenvironment is predominantly influenced by the activation of STAT3 and its interaction with NF-κB. In cancer and inflammatory cells, STAT3 activation is frequently driven by cytokines produced as a result of NF-κB signaling in the TME [181].

In addition, the mechanistic relationship between STAT3 and NF-κB is complex, multi-layered, and conditionally dependent [170, 182]. This activation is often facilitated by inflammatory cytokines, including IL-6, which are expressed as targets of NF-κB or STAT3-regulated genes [183–185]. Additionally, COX-2, a downstream mediator of NF-κB involved in tumor-promoting inflammation, serves as a STAT3 activator [162]. The NF-κB–IL-6 axis also activates STAT3 in response to cytokine mediators like IL-17, IL-23, and IL-21, which play critical roles in inflammation-driven tumorigenesis [186, 187].

The RELA-p50 heterodimer forms the core of the NF-κB complex, regulating inflammatory responses and mediating pro-oncogenic effects [188]. REL, another NF-κB family member, modulates the transcription of numerous immunostimulatory chemokines and cytokines linked to anti-tumor immune responses. Evidence suggests that EP300 (p300) acetylates proteins, promoting RelA nuclear accumulation [189]. Interestingly, STAT3 is essential for EP300-induced RelA acetylation, ensuring continuous activation of RelA in both cancerous and non-transformed cells within the TME [190]. Furthermore, NF-κB and STAT3 jointly regulate a range of inflammatory and oncogenic genes [191]. As critical pro-carcinogenic mediators, NF-κB and STAT3 suppress STAT1 and NF-κB-dependent immune responses, thereby undermining anti-tumor immunity [163].

Egusquiaguirre et al. [192] highlighted the intricate relationship between STAT3 and NF-κB signaling in breast cancer, revealing that STAT3 regulates the NF-κB pathway by targeting the *TNFRSF1A* gene. Similarly, Li et al. [193] demonstrated, using an in vitro model of tumor-derived DCs from patients with non-small cell lung cancer (NSCLC), that the TME alters DC functionality by simultaneously suppressing canonical NF-κB and STAT3 signaling, leading to dysregulated transcription of downstream genes [193]. Furthermore, Liu et al. [194] showed that in chronic lymphocytic leukemia (CLL), active STAT3 and RelA sustain Mcl-1/Bcl-xL anti-apoptotic protein expression and autocrine IL-6 production, rendering CLL cells resistant to conventional chemotherapy and apoptosis in vitro.

## Inflammatory biomarkers in Cancer diagnosis and prognosis

NF-κB is a pivotal pro-inflammatory signaling mediator that is often found excessively active in malignancies. Investigations have revealed that NF-κB stimulates numerous genes linked to acute inflammatory responses, including cytokines and co-stimulatory molecules that promote Tm cell activation and survival [195, 196]. It also regulates inhibitors of apoptosis proteins (IAPs), which impede both extrinsic (external) and intrinsic (mitochondrial) apoptosis mechanisms. Furthermore, IL-1 and TGF are known to induce sustained inflammation and contribute to genetic instability by generating ROS and impairing DNA repair processes. Pro-inflammatory mediators such as eicosanoids and prolactin, alongside other inflammatory agents, play considerable effects in cancer promotion driven by these factors [61, 197, 198].

The connection between cancer and chronic inflammation is well-established, with ample evidence supporting their strong correlation [61]. Chronic inflammation arises when the immune system fails to effectively detect and remove damaged or diseased cells that should undergo apoptosis or necrosis [199]. Several inflammatory agents are tightly correlated with the growth of precancerous damage, thereby triggering carcinogenesis [200]. Inflammatory mediators like IL-6 and TNF-α stimulate the proliferation of rapidly dividing cells, either directly as growth factors or indirectly by activating mitogen-activated protein kinases (MAPKs), such as Erk, c-Jun N-terminal kinases (JNK), and p38 [201]. These MAPKs further enhance gene transcription, which is crucial to the progression of the cell cycle.

### Measurement of C-Reactive protein

C-reactive protein (CRP), a key player in the innate immune response, is implicated in various biological events related to cancer progression [202]. CRP is transported to the liver *via* the angiotensin-converting enzyme receptor-2 (ACE2), where it exerts localized or systemic effects [203]. It can also initiate an inflammatory cascade. Interestingly, CRP exhibits dual roles in tumor biology, potentially promoting or inhibiting cancer cell killing. It protects against mononuclear cell cytotoxicity and antibody-mediated destruction while also contributing to chronic inflammation through NF-κB-derived gene products. This chronic inflammation supports tumor growth and systemic tissue damage [204–206].

Approximately 25% of glioblastoma patients exhibit elevated CRP levels, correlating with reduced overall survival. Blood CRP levels are commonly used to monitor inflammation and cancer progression in affected individuals [207]. Historically, elevated CRP levels were considered a predictive biomarker for immunotherapy responses in specific contexts [208]. However, this method was phased out in 2018 due to the preference of CRP to react with monomeric CRP (mCRP), which rapidly transitions into normal pentameric CRP (pCRP) [209].

In patients with advanced cancer, elevated CRP levels are commonly experienced in conjunction with elevated levels of IL-1β and TNF [210]. These patients may also experience reduced caloric intake, malnutrition, heightened oxidative stress, and impaired T cell telomere maintenance. CRP levels are widely recognized as systemic inflammation biomarkers and have been associated with poorer cancer outcomes [211]. Furthermore, elevated plasma CRP levels correlate with reduced progression-free and overall survival across various solid tumors, including glioblastoma multiforme, cervical cancer, anal cancer, and NSCLC, as confirmed by multivariate analyses [212].

## Pharmacological agents and approaches to addressing inflammation in Cancer treatment

Anti-inflammatory medications are not conventionally employed as primary treatments for cancer. However, current investigations have pointed to the critical role of inflammation in tumor progression and emphasized the molecular mechanisms underlying this event. These outcomes have sparked considerable attention in exploring the potential of anti-inflammatory medications to hinder or delay cancer development [213–219].

Chronic inflammation contributes to immune suppression and exacerbates cancer-associated inflammation, promoting cancer cell metastasis, tumor dedifferentiation, and resistance to chemotherapy [220]. These insights have positioned anti-inflammatory agents as promising adjuncts in cancer therapy regimens, offering a potential avenue to disrupt the inflammatory pathways that drive tumor progression.

### Common Anti-Inflammatory drugs for Cancer therapy

To regulate inflammation, oncologists frequently administer statins, corticosteroids, and NSAIDs. Although their mechanisms of action are distinct - corticosteroids induce alterations to gene transcription, statins affect NF-κB signaling and NSAIDs block COX enzymes - they all act together to inhibit inflammatory events in the TME.

#### NSAIDs

COX-2, a variant of the prostaglandin-endoperoxide synthase enzyme, is overexpressed in cancerous tissues and plays a pivotal role in inflammation and cancer cell proliferation [221]. NSAIDs have been investigated for their potential cancer-preventive effects, though clinical results remain mixed. For instance, Lu et al. [42] demonstrated that patients with chronic inflammatory bowel disease undergoing long-term anti-inflammatory therapies had a reduced likelihood of developing malignancies.

The presence of genetic variations in the *COX-2* and *NOS* genes is linked to ROS production within cells, causing DNA damage and potentially leading to cancer [222]. NSAIDs have been shown to inhibit cancer cell growth, disrupt prostaglandin synthesis, modulate growth factor pathways, suppress angiogenesis, enhance immune responses, and reduce pro-inflammatory signaling in tumor cells. These actions collectively reduce tumor-associated inflammation in the microenvironment.

Aspirin remains one of the most commonly prescribed NSAIDs for its significant cancer-preventive potential, as supported by numerous studies [223, 224]. Low-dose aspirin is particularly recommended for cancer patients due to its favorable safety profile. Data from the VICTOR cohort, involving 1,273 patients, showed that aspirin use at cancer diagnosis was associated with reduced cancer-related mortality, especially in colorectal cancer cases [225–229].

**Fig. 4:**
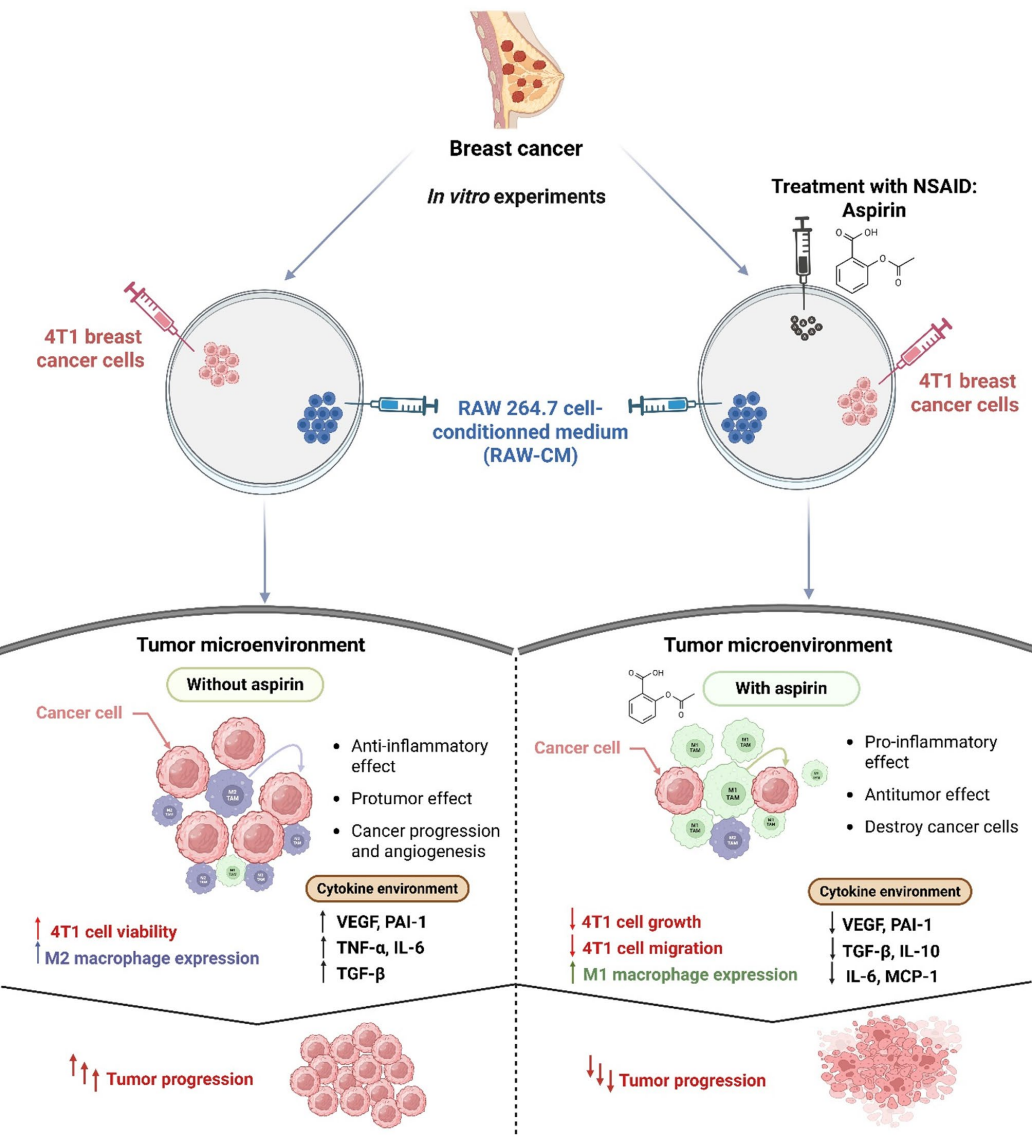
Schematic demonstration of the potential anti-cancer action of aspirin used simultaneously as an anti-inflammatory drug against 4T1 breast cancer in vitro. In an in vitro environment of 4T1 breast cancer cells, the infiltration of RAW264.7 macrophages upregulated the levels of VEGF, PAI-1, TNF-α, IL-6, and TGF-β, as well as the expression of M2 macrophages, resulting in tumor growth. Treatment with aspirin resulted in reduced generation of angiogenic and inflammation-associated cytokines VEGF, PAI-1, MCP-1, IL-6, IL-10, and TGF-β. Furthermore, treatment with aspirin resulted in upregulation of M1 expression and downregulation of M2 expression in macrophages, leading to a cross-talk in this microenvironment and hindered tumor progression. Treatment with aspirin significantly affected subtype M1/M2 macrophages and impaired the interplay between breast cancer cells (4T1) and RAW 264.7 cells by monitoring the generation of inflammatory and angiogenic signaling mediators. These findings indicate that aspirin may be a potentially valuable agent in the management of triple-negative breast cancer, as it reduces the tumor-promoting microenvironment. Abbreviations: IL-6, interleukin-6; IL-10, interleukin-10; TGF-α, tumor necrosis factor-α; TAM, tumor-associated macrophage; TGF-β, transforming growth factor-*β*; VEGF, vascular endothelial factor; plasminogen activator inhibitor-1; MCP-1, monocyte chemoattractant protein-1

Hsieh et al. [230] provided further evidence through in vitro studies demonstrating that aspirin suppresses TGF-β, IL-6, and MCP-1 in breast cancer cell lines (4T1) while modulating inflammation-related and angiogenic cytokine expression in macrophages and tumor cells. Aspirin promotes M1 macrophage polarization while inhibiting M2 polarization, limiting tumor progression [231] (Fig. 4).

Randomized controlled trials have also supported aspirin’s ability to prevent colorectal cancer, with outcomes influenced by patients’ age, height, and weight for reasons yet to be determined. Preclinical studies using animal models have demonstrated that aspirin reduces colonic dysplasia and polyp formation in genetically or chemically induced colon cancer. Additionally, aspirin inhibits the growth of cancer cells transplanted into normal or immunocompromised mice, supporting its potential as an adjunct in cancer therapy. Clinical studies further highlight aspirin’s ability to reduce cancer metastasis and improve survival rates when used before and after diagnosis [214, 232–234].

Celecoxib, another NSAID, has shown promising results in both preclinical and clinical research for various cancers, including breast, colon, neck, and prostate cancers [235]. For instance, combining celecoxib with chemotherapy in COX-2-positive gastric cancer patients significantly improved short-term clinical outcomes, disease-free survival (DFS), and progression-free survival (PFS) without notable side effects [236].

**Fig. 5:**
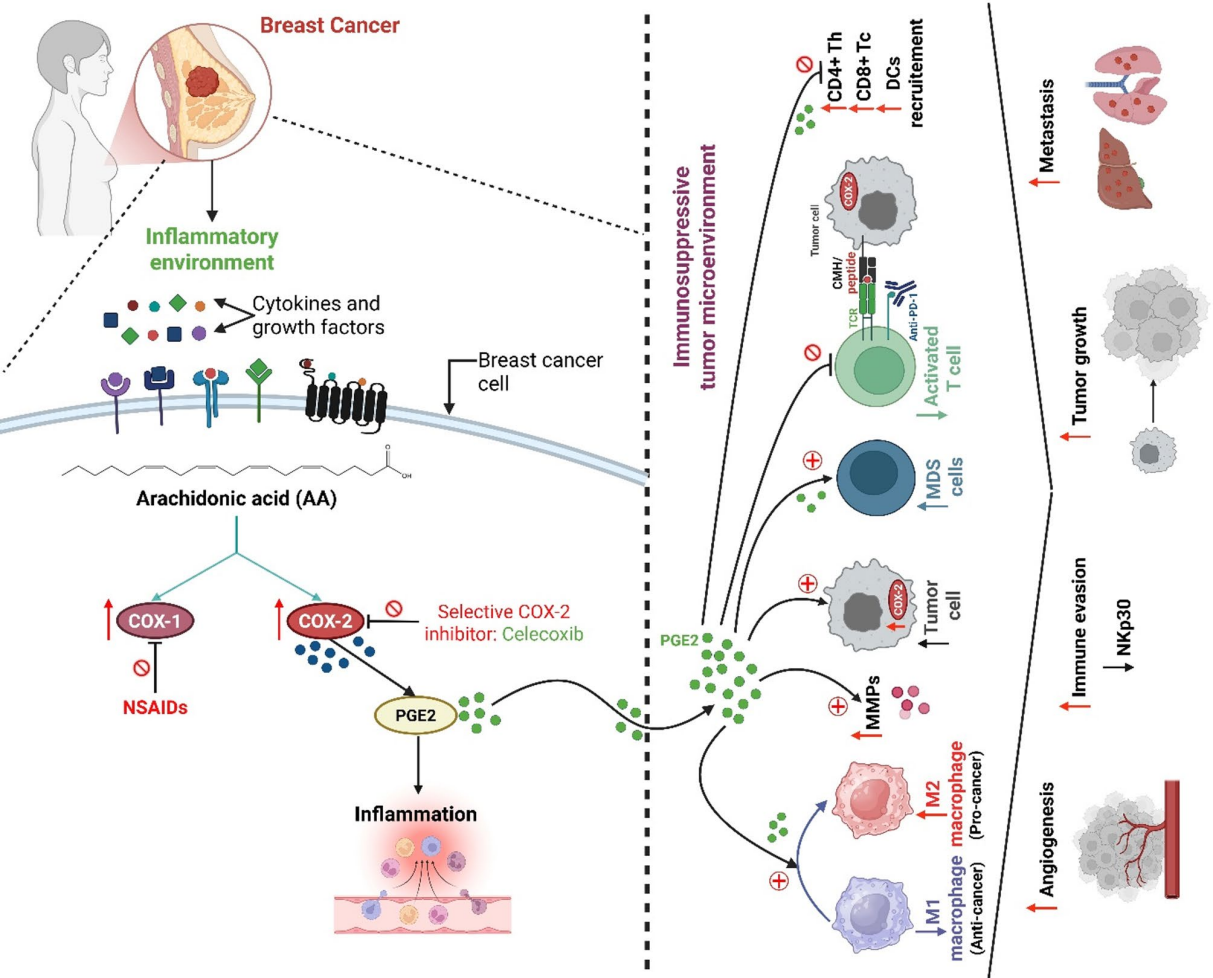
Proposal of the main molecular signaling pathways by which celecoxib exerts its anti-inflammatory action as a promising strategy in breast cancer therapy. By stimulating a wide range of immunosuppressive cells, the COX-2/PGE_2_ pathway promotes the sustainment of an immunosuppressive TME. The figure illustrates precisely how COX-2/PGE_2_ hyperactivity is involved in establishing an immunosuppressive TME, as evidenced by the signaling interplay between different cell types in the tumor landscape. Although celecoxib is well regarded for its ability to selectively inhibit COX-2, a key enzyme in the inflammatory mechanism, it has also attracted interest for its cancer-fighting properties, especially concerning cancer pathophysiology and metastasis. As a major mediator of the TME, PGE_2_ is reduced by celecoxib, which in turn plays a crucial role in controlling tumor biology. Celecoxib directly affects angiogenesis, essential for tumor growth, by inducing a reduction in PGE_2_ levels. Used alone or in combination with other chemotherapeutic drugs, selective inhibitors of COX-2 signaling such as celecoxib can be an effective partner in the fight against breast cancer. Abbreviations: PGE_2_, prostaglandin E2; COX, cyclooxygenase; MDSC, myeloid-derived suppressor cells; PD-1, programmed death-1; NK, natural killer; CD, cluster of differentiation; MMPs, matrix metalloproteinases; DCs, dendritic cells; NSAIDs, Non-steroidal anti-inflammatory drugs

In a Phase II trial, celecoxib improved cytological and serum biomarkers in women at high risk of breast cancer [237]. Recent studies have explored celecoxib’s ability to enhance paclitaxel-induced immunogenic cell death by inhibiting tumor-derived PGE_2_ production [238]. In a TNBC trial, combining celecoxib with paclitaxel DC maturation and T-cell-mediated immune responses, improved anti-tumor efficacy. Additionally, celecoxib inhibits COX-2 selectively, reducing PGE_2_ production, thereby attenuating VEGF-mediated angiogenesis. By diminishing PGE_2_-induced immunosuppression, celecoxib enhances immune responsiveness, suppresses metastasis, and reduces MMP activity in breast cancer [239] (Fig. 5).

Despite these promising findings, NSAID use is associated with potential adverse effects, including osteoporosis, metabolic disorders, mucosal damage, gastrointestinal ulceration, and bowel inflammation. Therefore, risk-benefit analyses are essential to evaluate the appropriateness of NSAIDs for cancer prevention and therapy [240].

#### Corticosteroids

Corticosteroids are among the most potent anti-inflammatory agents, capable of inhibiting cancer progression by reducing inflammation. These hormones exhibit direct cytotoxic effects on cancer cells, inducing cell cycle arrest and apoptosis. Moreover, they regulate the immune response and promote DNA repair [241–244].

Randomized controlled trials have demonstrated the efficacy of systemic corticosteroids in alleviating breathlessness in cancer patients, particularly those with pulmonary complications [245]. Dexamethasone is one of the corticosteroids frequently prescribed for cancer treatment, due to its marked immunosuppressive and anti-inflammatory properties. These drugs are often utilized to attenuate inflammation in the TME, control the adverse effects caused by chemotherapy, and manage tumor-related edema. By suppressing NF-κB and other pro-inflammatory transcription factors, corticosteroids reduce the release of cytokines (such as IL-1, IL-6, and TNF-α), thus altering the tumor’s immunological environment [246]. Accordingly, Samdaengpan et al. [228] recently reported that cancer patients treated with corticosteroids exhibited a lower seroconversion rate following COVID-19 vaccination, reflecting a dampened immune response. When combined with immunotherapy, this phenomenon highlights the systemic immunosuppressive effects of corticosteroids and underlines significant concerns about their potential impact on anticancer immune responses. Therefore, their use must be carefully balanced to prevent compromising therapeutic efficacy.

In another recent study, the combination of dexamethasone and tamoxifen was found to induce apoptosis, decrease the expression of E2F3 (a transcription factor) and SOX2 (sex-determining Y-box 2 region), and synergistically inhibit proliferation and migration of tamoxifen-resistant LCC-2 (TAMR-1) cells. This combination also caused S- and G_2_-M-phase arrest. These findings suggest that dexamethasone, in addition to its anti-inflammatory properties, could be a promising therapeutic approach to overcoming tamoxifen resistance by targeting E2F3/SOX2/Wnt signaling pathways [247].

Clinical studies have also shown that adding dexamethasone to lenalidomide and carfilzomib significantly improves PFS in patients with relapsed/refractory multiple myeloma (RRMM) [248]. Furthermore, xenograft experiments involving colorectal cancer, lung cancer, glioma, and breast cancer demonstrated that dexamethasone pretreatment enhanced the efficacy of carboplatin, gemcitabine, or their combination by 2- to 4-fold [249].

Katayama et al. [250] conducted a retrospective study involving 204 patients treated with corticosteroids and 139 controls who did not receive the treatment. Their findings indicated that corticosteroids did not impact the lifespan of end-stage cancer patients, as there was no substantial divergence between treatment and control groups regarding survival.

#### Statins

Statins are widely used to lower high blood cholesterol levels by inhibiting HMG-CoA reductase (HMGCR), the rate-limiting enzyme of the mevalonate pathway [251]. Due to their diverse therapeutic applications, statins have gained recognition as affordable, safe, and effective drugs in cancer treatment, particularly in combination with radiotherapy and/or chemotherapy, owing to their anti-inflammatory and anti-angiogenic properties [252]. Furthermore, in an in vivo study, the anticancer effects of liposomal 5-fluorouracil were significantly enhanced by liposomal simvastatin in murine C26 colon carcinoma models [253]. Additionally, statins have demonstrated the ability to temporarily modulate the surface expression of prostate-specific membrane antigen (PSMA) and epidermal growth factor receptor (EGFR) on tumor cells. This modulation improves the tumor-binding avidity of monoclonal antibodies, including panitumumab, huJ591, and cetuximab, thereby amplifying their anticancer effects [253]. In addition, by inhibiting the mevalonate pathway, statins have been shown to attenuate radiation resistance in head and neck carcinomas, highlighting the pathway’s potential as a key target in preventing the development of such resistance [254]. Moreover, statins suppress cancer cell growth by modulating critical signaling pathways, such as mammalian target of rapamycin (mTOR), mitogen-activated protein kinase (Ras-MAPK), Erk1/2, NF-κB, and phosphoinositide 3-kinase (PI3K)/Akt [252, 255]. Statins also exert anti-angiogenic effects by regulating VEGF, MMP9, IL-6, and IL-8. They impact Rho family protein delocalization, which inhibits angiogenesis and metastasis, as demonstrated in vitro using CCL17-mediated colorectal cancer models [256]. Furthermore, statins reduce malignant stem cell markers, thereby limiting cancer progression [256] ; [257, 258].

Recent in vitro research by Göbel et al. [259] identified a responsive reparatory feedback mechanism involving the HMGCR/sterol regulatory element-binding protein-2 (SREBP-2) pathway, which plays a critical role in statin resistance. This was observed in human prostate cancer cell lines, including C4-2B, PC3, LNCaP, and DU-145 [259]. Additionally, statins have emerged as a valuable tool for reducing cancer risk in individuals with systemic inflammatory diseases. By addressing the underlying inflammatory processes, statins offer a targeted approach to cancer prevention, responding to the social and personal challenges associated with these conditions [260].

### Targeted cytokines and inflammatory pathways inhibitors as a treatment strategy

#### Anti-TNF-α and anti TNF-α-NF-κB pathway

Infliximab, adalimumab, and golimumab are three examples of monoclonal antibodies that target TNF-α and are designed to block the conventional TNF-α signaling pathway. Phase I and II clinical trials have evaluated these drugs; infliximab has shown good tolerability in patients presenting advanced malignancies and exhibited no dose-limiting side events [100]. Furthermore, Guo et al. have shown in their recent study that inhibition of the TNF-α/TNFR2 signaling system significantly delays colorectal cancer growth and increases the efficacy of anti-PD1 immunotherapy. The main mechanisms involved are reduced TNF-α secretion and reduced CCR8 + Tregs, known to promote immunosuppression in the TME. The tumor is more responsive to immune checkpoint blockade when these immunosuppressive cells are reduced. The clinically used TNF-α inhibitors etanercept and infliximab are presented in the study as viable options for reorientation in conjunction with anti-PD1 therapy, providing a promising approach to improving immunotherapy outcomes in colorectal cancer patients [261]. TNF-α processing inhibitor-1 (TAPI-1) is a well-recognized metalloproteinase suppressor with potential anti-inflammatory properties. Whether it has anticancer properties on esophageal squamous cell carcinoma (ESCC) has yet to be discovered. According to Gao et al., TAPI-1 significantly reduces the growth of ESCC cells. Key findings show that TAPI-1 reduces ESCC cell migration and invasion, induces apoptosis, and decreases cell proliferation. These effects are achieved mechanistically by inhibiting the NF-κB signaling system, which is frequently active in ESCC and associated with inflammation and tumor growth. According to the study, TAPI-1 could be used as a treatment for ESCC as it inhibits inflammatory signals that promote tumor growth and targets the NF-κB pathway [262]. Crucially, long-term use of NF-κB suppressors can induce immunological deficits. Therefore, the optimal application of these inhibitors in cancer therapy should be a brief intervention. A suitable NF-κB inhibitor should only interfere with the NF-κB pathway, without affecting other signaling pathways [263].

#### Anti-IL6 and Anti-IL6-STAT3 pathway

Numerous immunological cells like T lymphocytes, B lymphocytes, monocytes, macrophages, and tumor cells within the TME all produce the pleiotropic cytokine IL-6. Ras-induced IL-6 production facilitates the active promotion of tumor growth by the TME. IL-6 has both pro- and anti-inflammatory properties, which greatly enhance tumor growth [264]. Olokizumab, siltuximab, sirukumab, MEDI5117, and clazakizumabare are anti-IL-6 monoclonal antibodies that block signal pathways [263]. The main results of the recent study conducted by Haq et al. demonstrate that IL-6R immunotherapy, combined with targeting the MCT-1/IL-6/CXCL7/PD-L1 signaling axis, significantly reduces the risk of metastasis and relapse in TNBC. The study revealed that this combined approach decreases immune evasion, tumor-promoting inflammation, and the likelihood of metastasis. In particular, IL-6R blockade by drugs such as tocilizumab and anti-PD-L1 therapies like atezolizumab enhances antitumor immunity and slows tumor growth, offering a viable treatment strategy to improve outcomes in TNBC [265]. Moreover, key highlights from a phase I trial by Dijkgraaf et al. (2015) show that tocilizumab combined with carboplatin/doxorubicin and interferon-α2b-based chemotherapy was effective and well sustained in patients with recurrent epithelial ovarian cancer. By lowering CRP levels, the drug effectively reduced IL-6 signaling and altered the immunological milieu without compromising chemotherapy efficacy [266]. On the other hand, Angevin et al. noted that siltuximab, another anti-IL-6 monoclonal antibody, proved well tolerated and biologically effective in patients with solid tumors that had progressed. The maximum tolerated dose, safety profile, and pharmacokinetics of siltuximab were determined in the Phase I/II dose-escalation trial, with some patients showing signs of disease stabilization. Treatment resulted in lower CRP levels, demonstrating that the IL-6 pathway is inhibited. Despite modest objective tumor responses, the trial has laid the foundations for future research into IL-6 blockade as a therapeutic approach for solid tumors, particularly when combined with other therapies [267]. In a Phase I-II clinical trial, prostate cancer patients treated with siltuximab showed lower levels of activated STAT3 and MAPK [268]. Also, after receiving siltuximab, over 50% of patients with metastatic renal cell carcinoma showed signs of disease stabilization in another Phase I-II trial [269]. According to the key findings of the research by Brooks et al. (2016), IL-6 trans-signaling is essential for advancing KRAS-induced lung carcinogenesis. In KRAS mutant models, the researchers showed that IL-6 trans-signaling, which is mediated by the soluble IL-6 receptor (sIL-6R), accelerates lung tumor development by enhancing inflammation, tumor cell proliferation, and immune evasion. The specific inhibitor sgp130Fc significantly reduced tumor burden and inflammatory cell infiltration by blocking IL-6 trans-signaling. These results imply that a promising therapeutic approach for lung tumors with KRAS gene mutations could be to specifically target IL-6 trans-signaling [270]. In addition, Hu et al. (2024) have recently demonstrated that the IL-6R inhibitor tocilizumab can effectively disrupt the IL-6-STAT3-C/EBPβ-IL-6 positive feedback loop in TAMs. By inhibiting IL-6 receptor signaling, tocilizumab reduced the downstream expression of C/EBPβ and activated STAT3, thereby decreasing IL-6 production and weakening the loop that promotes EMT and lung adenocarcinoma metastasis. According to these results, tocilizumab is a promising therapeutic option for lung cancer that targets the tumor-promoting microenvironment, as it has the ability to inhibit tumor growth and inflammation-induced metastasis [271]. Highlights of Shi et al.‘s 2024 investigation indicate that bazedoxifene, a selective estrogen receptor modulator, effectively blocks IL-6/GP130-mediated activation of STAT3, a key signaling pathway involved in cancer development. According to the study, bazedoxifene inhibited the growth, migration, and survival of cancer cells across various tumor types, including malignant pancreatic and breast tumors. By inhibiting the IL-6/GP130/STAT3 pathway, bazedoxifene disrupts inflammation and signaling that promotes tumor growth. These results support its potential reapplication as an anti-cancer drug, particularly in malignant tumors triggered by abnormal IL-6 signaling [272]. Furthermore, the major aims identified in the research by Méndez-Clemente et al. (2022) are to demonstrate that migration, invasion, and proliferation of prostate cancer cells are strongly reduced in vitro when the IL-6/IL-6R/STAT3 axis is doubly inhibited by stattic, a STAT3 inhibitor, and tocilizumab, an IL-6R monoclonal antibody. The combined therapy demonstrated a synergistic effect in reducing the important processes involved in tumor growth, surpassing the performance of either drug alone. Based on these results, a promising therapeutic approach for advanced prostate cancer could be to focus on both IL-6 signaling and STAT3 activation [273]. Hence, there is limited evidence of the potential benefits of single-agent clinical trials for solid tumors, although preclinical research is targeting IL-6-STAT-3 signaling pathways. In unselected patient populations, this highlights how difficult it is to achieve significant prognostic changes simply by suppressing IL-6. It is essential to develop effective combination therapies and find reliable biomarkers to predict response to therapy.

#### Anti-CCL2/CCR2 Axis

Recruitment and survival of myeloid cells, such as inflammatory monocytes, TAMs, and MDSCs, depend on the CCL2/CCR2 axis, a potent pro-inflammatory chemokine. Consequently, suppression of the CCL2/CCR2 axis has been investigated as a therapeutic approach to modify immunosuppressive EMT and trigger anticancer immunity [15]. Carlumab (CNTO 888), a human anti-CCL2 mAb, demonstrated transient CCL2 suppression and anticancer efficacy in patients with solid tumors in the first-ever human clinical trial. Carlumab did not demonstrate significant anticancer activity as a single drug in a Phase II study, although it was used safely in patients with metastatic CRPC [274]. In another Phase I trial (NCT01204996), patients with solid tumors were evaluated with carlumab in conjunction with four different chemotherapy regimens. No long-term tumor response was observed in any of the patients tested, and although carlumab was well tolerated when used in combination with conventional chemotherapies. The most frequent drug-related grade 3/4 adverse events were neutropenia for gemcitabine and docetaxel [275].

In patients receiving first-line treatment of metastatic pancreatic ductal adenocarcinoma (PDAC), PF-04136309, a small-molecule CCR2 antagonist, was well tolerated and showed early antitumor activity when combined with gemcitabine and nab-paclitaxel, according to the main results of a Phase 1b study conducted by Noel et al. (2020). CCR2⁺ monocytes, which are linked to immunosuppressive TMEs, were successfully reduced by combination therapy, indicating that CCR2 inhibition may improve immune response and chemotherapy efficacy [276].

Similarly, Nywening et al. (2016) found that CCR2 inhibition by PF-04136309, when combined with FOLFIRINOX chemotherapy, is safe and well tolerated in patients with locally advanced and borderline resectable pancreatic cancer. By effectively reducing CCR2⁺ monocytes and TAMs, PF-04136309, a small-molecule CCR2 antagonist, reduced immunosuppression and stimulated T-cell infiltration into the TME. In the treatment of pancreatic cancer, this immune modulation was linked to a better pathological response and a higher-than-expected surgical resection rate, confirming PF-04136309’s potential as an adjuvant to chemotherapy [277].

Recently, Modak et al. (2024) found that acute myeloid leukemia (AML) resistance to MEK (also known as Mitogen-activated protein kinase kinase and MAP2K) inhibitors can be successfully overcome by focusing on the CCL2/CCR2 signaling pathway. Leukemia cells responded better to MEK inhibitor treatment when this pathway was blocked, indicating a synergistic impact. The use of CCR2 inhibitors, including PF-04136309 and BMS-813,160, as potential drugs to suppress CCL2/CCR2 signaling, is explicitly highlighted in the study. These drugs restore the efficacy of MEK inhibition in resistant AML by disrupting the pro-survival milieu that is powered by CCL2-mediated monocyte and macrophage migration [278].

#### JAK inhibitors

Several JAK inhibitors are currently being researched and clinically evaluated for the treatment of cancers linked to inflammatory diseases. By specifically inhibiting JAK, small molecule tyrosine kinase inhibitors such as pacritinib, AZD1480, ruxolitinib, and tofacitinib prevent STAT-3 phosphorylation. These JAK inhibitors have shown considerable promise as potent anti-cancer drugs in clinical trials, inhibiting the growth of a variety of solid tumors, such as breast, lung, colorectal, and brain tumors [279]. The potential of second-generation JAK2 inhibitors as novel therapeutic agents for advanced prostate cancer is highlighted by Beinhoff et al. in their findings. This is especially true as these drugs can specifically target the JAK2/STAT3 signaling pathway, which is crucial for tumor progression, immune evasion, and treatment resistance. These newer substances minimize off-target effects while offering greater selectivity and potency than first-generation inhibitors. The study highlights a number of promising drugs, such as NS-018 (Ilginatinib), fedratinib, pacritinib, and momelotinib, all of which have demonstrated positive preclinical results in reducing cancer cell proliferation and spread [280]. Ruxolitinib, a JAK1/JAK2 inhibitor, is beneficial in treating patients with atypical chronic myeloid leukemia (aCML) and chronic neutrophilic leukemia (CNL), particularly those with CSF3R mutations, according to the main finding of the study by Dao et al. (2020). Most importantly, by blocking pro-inflammatory cytokine signaling via the JAK/STAT pathway, Ruxolitinib also markedly reduced inflammation-related symptoms such as fever, night sweats, and exhaustion. Based on these results, ruxolitinib shows promise in treating inflammatory symptoms and disease burden in people with CNL and aCML [281]. Ruxolitinib effectively suppresses CRP expression in inflammatory human hepatocytes, according to a study conducted by Febvre-James, Lecureur and Fardel. The study demonstrated that by blocking the JAK/STAT3 signaling pathway, which regulates inflammatory responses in hepatocytes, ruxolitinib significantly reduced IL-6-induced CRP expression. These results demonstrate the significant anti-inflammatory effects of ruxolitinib and provide evidence for its possible application in diseases characterized by excess IL-6-induced inflammation, such as inflammation-related tumors [282]. Another JAK1/JAK2 inhibitor, AZD1480, is being considered as a possible treatment to overcome cisplatin resistance. It targets the JAK/STAT signaling system, which is linked to chemoresistance, including to cisplatin, and is frequently triggered in tumor development by inflammation. According to the study, AZD1480’s reduction of JAK/STAT3 signaling can decrease immune evasion, tumor-promoting inflammation, and survival signaling, thereby increasing the sensitivity of cancer cells to cisplatin in an animal model of HPV-associated head and neck squamous cell carcinomas (HNSCC) [283].

In their Phase 1b study (2022), Padda et al. evaluated the effects of the combination of the EGFR tyrosine kinase inhibitor erlotinib and the JAK1/JAK2 inhibitor momelotinib in EGFR-mutated, tyrosine kinase inhibitor (TKI)-naïve metastatic NSCLC patients. In fact, erlotinib and momelotinib administered together proved safe, acceptable and had controllable side effects. A few people stabilized their disease and achieved partial responses, suggesting a possible synergistic action. Targeting both EGFR signaling and JAK/STAT-mediated inflammation and resistance pathways to delay or overcome resistance to EGFR inhibitors is therefore rationale [284]. By inhibiting the JAK-STAT signaling pathway and thereby decreasing the activity of important pro-inflammatory cytokines such as IL-2, IL-6, IL-12, IL-23, and interferons, the JAK1/JAK3 inhibitor tofacitinib demonstrates therapeutic efficacy in treating inflammatory disorders [285]. However, the same mechanism that inhibits immune system activation may hinder immune surveillance against newly developed tumor cells. According to Bezzio et al.‘s 2023 systematic review and meta-analysis, patients receiving tofacitinib had a slightly higher risk of developing cancer overall. They revealed a specific correlation between lung cancer and lymphoma, particularly in individuals with predisposing factors such as advanced age or a history of smoking [286]. Previous pooled analyses of clinical trials showing comparable patterns corroborate these findings [287, 288]. Recently, Zhang et al. assessed the safety and efficacy of jaktinib, a novel JAK inhibitor, in treating patients with myelofibrosis who had relapsed or failed to respond to ruxolitinib in their Phase II study, published in 2023. Key findings indicated that jaktinib demonstrated encouraging clinical action, with improvements in symptom scores and a significant percentage of patients achieving a ≥ 35% reduction in spleen volume. Additionally, jaktinib exhibited a reasonable safety profile, was generally well-tolerated, and most side effects were hematological and transient. These results highlight an important unmet clinical need: jaktinib could serve as a viable therapeutic alternative for myelofibrosis patients who no longer respond to ruxolitinib [289]. The potential of pacritinib, a JAK2/FLT3 inhibitor, to block the JAK/STAT signaling pathway in patients with resistant metastatic colorectal cancer was examined in the 2017 study by Regenbogen et al. Initial safety and efficacy were to be assessed in this trial. Key findings showed that although pacritinib was well tolerated, there was little clinical activity in this group of patients who had received many previous treatments. The study revealed that STAT3 signaling was effectively inhibited, despite limited antitumor benefits. This suggests that combination techniques or use in biomarker-selected populations, where JAK/STAT-induced inflammation plays a greater role in tumor growth, may be used [290]. Similarly, using preclinical models of AML, Novotny-Diermayr et al. (2012) examined the oral HDAC inhibitor pracinostat (SB939) in conjunction with the JAK2 inhibitor pacritinib (SB1518). The main findings revealed that the combination therapy acted in concert with the other drug to induce greater anti-leukemic effects than either alone. This synergy is mechanistically linked to the dual inhibition of cancer-promoting pathways, such as the epigenetic modification of pracinostat and the suppression of inflammatory signaling by pacritinib through inhibition of the JAK2/STAT3 pathway. Activation assays of STAT3 and levels of pro-inflammatory cytokines, which are linked to immune evasion and AML progression, were markedly reduced by treatment. Based on these results, it may be possible to target both the inflammatory and carcinogenic aspects of AML by combining HDAC and JAK2 inhibitors [291].

### Omics technologies involvement in managing inflammation in Cancer

Pro- and anti-inflammatory imbalances within tumors can be precisely addressed through innovative technologies that combine lipidomics, spatial transcriptomics, and immunomonitoring on a cellular scale [292]. In light of the endogenous specialized pro-resolving mediators (SPMs) and their strong bioactivities in reducing inflammation, as well as the link between inflammation and cancer, the mechanisms of SPMs in cancer have also attracted interest and exploration [293]. Polyunsaturated fatty acids (PUFAs), primarily omega-3s, are the source of SPMs, a unique class of bioactive lipids that actively monitor the resolving phase of inflammation without altering the immune response. SPMs, including resolvins (D, Dp, and E series), protectins, maresins, and lipoxins, facilitate the removal of inflammatory cells, reduce neutrophil infiltration, enhance macrophage phagocytic activity (efferocytosis), and promote tissue repair, whereas standard anti-inflammatory drugs inhibit immune functions [294]. Resolvins (EPA (eicosapentaenoic acid) and DHA (docosahexaenoic acid)), protectins, and maresins (DHA), along with lipoxins (arachidonic acid), serve as precursors for each fatty acid family. They exert their effects by binding to specific G protein-coupled receptors (GPCRs), such as GPR32 and ChemR23. SPMs are vital for restoring homeostasis after acute stress, as they suppress pro-inflammatory cytokines like TNF-α and IL-6 [295]. Although understanding of the mechanisms underlying inflammation and cancer is growing, the potent anti-inflammatory effects of SPMs raise the possibility that they may regulate cancer development. There are several different mechanisms by which SPMs modulate inflammatory TME [296]. Resolvin D1 (RvD1) and lipoxin A4 (LXA4) can reduce angiogenic phenomena by binding to the formyl peptide receptor-like 1 (FPRL1) through VEGF suppression. However, by switching from the M1 to the M2 phenotype, macrophages can regain their anti-tumor properties thanks to the interaction between LXA4 and these cells [297]. In a similar context, RvD2 enhances phagocytosis, infiltration, proliferation, and survival of M2 cells, while decreasing pro-inflammatory cytokines such as IL-6, MPC1, CXCL10, and TNF in a variety of immune cell types present in the TME. Moreover, anti-tumor immune cells such as Tregs and neutrophils can be stimulated by LXA4, which may increase their antineoplastic activity or chemotaxis towards the tumor, while pro-tumor cells such as Breg lymphocytes can be inhibited. Thus, RvD1 and LXA4 interact with receptors on the surface of cancer cells to directly reduce key pro-tumor pathways such as invasion, metastasis, and EMT.

 [293, 294, 298, 299]. In their recent investigation, Soundararajan et al. performed an integrative analysis of the colorectal cancer landscape. They combined lipidomics, single-cell RNA sequencing, and spatial transcriptomics. Higher amounts of arachidonic acid derivatives, including leukotrienes and 5-HETE, were found to be indicative of a marked pro-inflammatory lipid profile in colorectal cancer. A significant lack of pro-resolving lipid mediators, such as resolvins, protectins, and lipoxins (LXA4, LXB4), was observed in conjunction with this pro-inflammatory state. Given that the prostaglandins PGE_2_ and PGD_2_ normally govern this process, the study attributed this imbalance to an altered class switch of lipid mediators, which was observed to be reduced in tumor tissue. These findings were corroborated by gene expression analyses, which revealed a down-regulation of pro-resolution enzymes (e.g. ALOX15, EPHX1) and an increase in pro-inflammatory enzymes (e.g. ALOX5, ALOX5AP). Tumor-associated macrophages have been identified as important contributors to the pro-inflammatory environment using single-cell studies and spatial transcriptomics [300]. Advanced omics technologies, combined with further research into SPMs, might offer enormous potential for designing novel tactics that would efficiently reduce the inflammation that promotes tumor growth, and simultaneously boost immune surveillance.

### Artificial intelligence (AI) as a tool for resolving Inflammation-Induced Cancer

The use of AI has grown considerably in recent years. Medical imaging assessment is becoming increasingly standardized through the use of AI-based computer-aided diagnosis (CAD) systems, which integrate machine learning (ML), deep learning (DL), and artificial neural networks (ANN). This is due to the development of deep learning and the availability of large imaging databases [301]. AI models have been employed to characterize immune and inflammatory cell cancer models [302]. A deep learning-based image analysis method was developed by Linder et al. (2019) to identify and measure tumor-infiltrating lymphocytes (TILs) in testicular germ cell tumors (TGCTs). This methodology provides an unbiased and reproducible approach to TIL assessment, exhibiting high concordance with expert pathologists’ evaluations. The pathological study of TGCTs can greatly benefit from this automated method, which can improve prognostic assessment and advance knowledge of the TME. The results show that artificial intelligence holds great promise for improving diagnostic accuracy and helping clinicians make decisions about cancer treatment [303]. Aprupe et al. designed a deep convolutional neural network (CNN) model in their study to reliably and accurately quantify immune cell biomarkers in the lung cancer microenvironment based on supervised learning [304]. On the other hand, most attempts to elucidate the mechanism underlying Apolipoprotein AI (Apo-AI) anti-inflammatory and anticancer activities have focused on how Apo-AI interacts with the scavenger receptor class B type (ISR-BI), ATP-binding cassette transporter (ABCA1), and ATP-binding cassette transporter G1 (ABCG1) receptors. Some studies have attempted to establish a link between ABCA1 and epithelial-mesenchymal transition in breast cancer, while others have demonstrated that mice lacking ABCG1 and ABCA1 showed reduced growth of tumors derived from subcutaneously transplanted melanoma or bladder carcinoma cells when subjected to a “Western-style” diet [305]. Furthermore, Guan et al. in their recent study used bioinformatics and machine learning techniques to analyze the recombinant protein phospholipase A2 group IB (PLA2G1B) for putative molecular targets involved in cancer development. According to their research, PLA2G1B could play a crucial role in regulating pathways linked to immunological response and tumor growth. The study identified a number of potential targets for therapeutic intervention by combining gene expression data and predictive modeling [306].

Given the critical role TLR4 plays in pro-inflammatory responses and the fact that traditional drug design methods are costly, time-consuming, and laborious, new TLR4 modulators discovered through AI and computer-aided drug design hold great promise and have demonstrated promising insights, particularly for treating inflammatory diseases related cancer [307]. Besides, compared to small molecules, anti-inflammatory peptides (AIPs) have proven to have greater therapeutic potential against cancer induced by inflammatory processes, thanks to their excellent selectivity and reduced toxicity. In this context, machine learning might play an essential role in peptide prediction [308]. In another recent study, for patients with papillary thyroid cancer (PTC), a deep learning radiomic signature of inflammation (DLRI) provides a reliable non-invasive method to assess prognosis and to direct anti-inflammatory traditional Chinese medicine (TCM). The model’s ability to predict molecular subtypes of PTC could help assess a patient’s prognosis and response to anti-inflammatory therapy [309]. Moreover, AI holds great promise for increasing the accuracy of diagnosis of skin lesions in relation to skin cancer. Indeed, the investigators identified that people suffering from atopic dermatitis, a long-term inflammatory skin condition, may be more likely to develop cancerous skin lesions. Incorporating AI technology tools into the area of dermatology could be a beneficial strategy, particularly for patients with atopic dermatitis or other chronic inflammatory conditions [310]. Regarding AI and inflammation-related colorectal cancer, Ji et al. recently used machine learning to assess the effect of dietary fiber on colorectal cancer patients and found that higher fiber intake was significantly linked to better clinical results, with a notable finding that patients with higher fiber intake had lower levels of inflammatory markers like IL-6 and CRP. Machine learning models identified correlations between fiber-rich diets and favorable prognostic factors, indicating that dietary fiber may contribute to controlling inflammation and enhancing survival [311]. Presently, it is well established that AI enables data-driven decision-making, resulting in more rapid drug discovery and development while reducing failure rates. Nevertheless, the practical use of these designs is hampered by a number of issues, notably the necessity of in-depth validation studies and the need to address legislative restrictions. Anti-inflammatory and resolving regimens are essential for affecting the tumor microenvironment, their interplay with immunotherapeutic strategies requires particular consideration, as will be discussed in the next section.

### Immunotherapeutic approaches

A key component of contemporary oncology, immunotherapy is used in addition to traditional anti-inflammatory drugs such as corticosteroids and NSAIDs. Although not specifically anti-inflammatory, these methods rely on immune modulation in the TME and interplay with inflammatory signaling pathways [312]. Immunosuppressive cell populations significantly influence the TME. One promising clinical strategy involves inhibiting the infiltration and activity of immune-suppressive cells within the TME [313, 314]. While definitive treatments remain elusive, attempts to combine chemotherapy with molecular targeting agents have shown some promise. In particular, using immunosuppressive cell-targeting drugs alongside immune checkpoint inhibitors (ICIs) has yielded synergistic effects, enhancing anti-tumor efficacy [315, 316]. These combinations aim to maximize therapeutic impact while minimizing systemic and on-target side effects.

Inflammation is a key contributor to tumor development [317]. Leveraging anti-inflammatory drugs to specifically target this process offers innovative therapeutic pathways [318]. For instance, pro-inflammatory proteins in the TME can impede chemotherapy efficacy and stimulate the growth of new blood vessels. Overexpression of NSR1 in glioma cells can confer chemotherapy resistance, whereas NSR1 inhibition has been shown to suppress inflammatory molecule expression, curtail tumor growth, and enhance chemotherapy sensitivity [319]. These results emphasize the importance of targeting inflammation to drive therapeutic advancements in clinical oncology. Nonetheless, further strategies are necessary to refine combination therapies and address the immunosuppressive milieu in the TME.

In recent years, therapies targeting immune inhibitory cells or immune checkpoint pathways have gained traction. Blocking negative immune checkpoints using inhibitors of CTLA-4 and programmed death-ligand 1 (PD-L1)/programmed death 1 (PD-1) has proven effective for many cancers, either as monotherapy or in conjunction with traditional antitumor treatments [320–322]. However, their effectiveness has been inconsistent, likely due to the dense immunosuppressive milieu in tumors. This milieu, composed of immune-suppressive cells, Tregs, cytokines, and chemokines, contributes to variable drug responses across patients [323, 324].

Emerging therapies have drawn attention to the role of anti-inflammatory agents in cancer treatment. Evidence supports the use of these agents to repolarize macrophages, decrease MDSCs, and inhibit cytokines involved in cancer cell proliferation. These approaches show promise as neoadjuvant therapies to disrupt cancer-associated inflammatory signaling [325–327]. MDSCs, which are immature myeloid cells with immunosuppressive benefits, have a crucial effect on the TME and are linked to tumor progression, metastasis, and relapse [328] ; [329].

Targeting MDSCs offers the potential to enhance immunotherapy by countering cancer-induced immunosuppression [330]. MDSCs, in their immature state, exhibit suppressive properties but can differentiate into mature myeloid cells, like macrophages or DCs, thereby reducing their immunosuppressive activity [331]. All-trans-retinoic acid (ATRA), a vitamin A derivative, has demonstrated the potency to mediate MDSC differentiation into macrophages and DCs by inhibiting retinoic acid signal transduction in cancer patients and animal models [332]. ATRA treatment has also been demonstrated to diminish ROS levels in MDSCs *via* activation of the ERK1/2 signaling pathway [333].

### Other innovative therapeutic strategies: toward the targeted resolution of inflammation in Cancer

Currently, several new approaches have emerged with potential outcomes in regulating cancer-related inflammation, complementing standard and targeted anti-inflammatory therapies. These strategies include advanced delivery systems or multivalent drugs designed to target the TME or inflammatory cytokines.

Nanoparticle-based therapies have also gained attention. Prodrugs attached to nanoparticulate albumin particles have demonstrated efficacy in preclinical investigations [334–336]. Studies indicate that radiolabeled monoclonal antibodies (mAbs), along with their unlabeled counterparts, accumulate in tumors at levels comparable to polyethylene glycosylated nanoparticles. These outcomes highlight the potency of mAbs and nanobodies in cancer diagnosis and therapy [337, 338]. Bispecific antibodies, designed to detect two distinct targets, have shown efficacy in certain breast cancer cases by targeting phosphorylated epitopes that trigger kinase autophosphorylation in live cells [200].

Other innovative approaches include the application of DNA-interacting agents, such as Z-asymmetrical inter-strand crosslinking compounds, which exhibit potent anticancer properties due to their exceptional physical and toxicological characteristics [339, 340].

Finally, propranolol, a non-selective β-adrenergic receptor blocker, has shown promise in reducing lung cancer metastasis by lowering IL-6 levels. The C-reactive protein adsorber column (CAC) has effectively reduced malignant pleural effusion in cancer patients by removing circulating cytokines such as TNF-α and IL-6 [341–344].

In summary, inflammation is a well-known risk factor contributing to the occurrence of various cancers, as it involves intricate interactions between the immune system, TME, and inflammatory processes that foster tumor progression [62]. Over the past two decades, numerous investigations have highlighted a substantial association between inflammation markers—either analyzed individually or as part of comprehensive multi-biomarker scores—and a higher risk of cancer occurrence and fatality [337].

Furthermore, it has been postulated that the specific immunological and inflammatory profiles present at baseline or during the administration of anti-cancer therapies significantly influence treatment responsiveness or resistance. This underscores the critical role of an individual’s immune response and inflammatory markers in shaping therapeutic outcomes. By deeply understanding and analyzing these profiles, healthcare providers can fine-tune therapeutic strategies, personalizing them to achieve optimal efficacy while minimizing resistance. This paradigm shift has the potential to revolutionize cancer treatment and provide the basis for more individualized and precise therapeutic interventions that improve patient prognoses. Moreover, inflammation not only accelerates cancer progression but also acts as a prognostic indicator, correlating with adverse clinical outcomes. Notably, patients with concurrent cancer and chronic inflammatory diseases (CIDs) tend to experience poorer prognoses than those without such comorbidities [345].

## Main discoveries and results regarding Anti-Inflammatory phytochemicals in Cancer therapy

Numerous studies have highlighted the mechanistic interactions between phytochemicals and cancer hallmarks, targets, or pathways (Table 1) [346–348]. These interactions frequently involve the regulation of gene expression to block the initiation and progression of cancer, such as breast cancer. Compelling evidence underscores the role of cancer-related inflammation in every stage of the carcinogenesis process, encompassing proliferation, survival, angiogenesis, invasion, and metastasis. Self-sustaining inflammation develops as cancer cells undergo metamorphosis, driven by internal and external alterations in the microenvironment of preneoplastic tissues and tumor sites [61].

Persistent inflammation facilitates the initiation, progression, and survival of tumors even after chemotherapy and radiotherapy. This phenomenon is linked to the involvement of immunosuppressive chemokines, cytokines, and the dysregulated behavior of innate and adaptive immune cells within the context of unresolved inflammation. Conversely, tumor-resolving inflammation correlates with improved outcomes, including reductions in circulating cytokines and angiogenic factors following treatment. This process fosters a dynamic microenvironment marked by tissue repair and the presence of mediators such as IL-1, IL-10, and IL-7, which bolster the immune response and enhance therapeutic efficacy while mitigating tumor regrowth. Successful tumor resolution is typically associated with the long-term stabilization of immunologic memory [61].

The unresolved inflammatory state within different tissues serves as a predictive marker for patient treatment responses and provides a basis for developing strategies to suppress primary, relapse, and metastatic cancer progression.

**Fig. 6:**
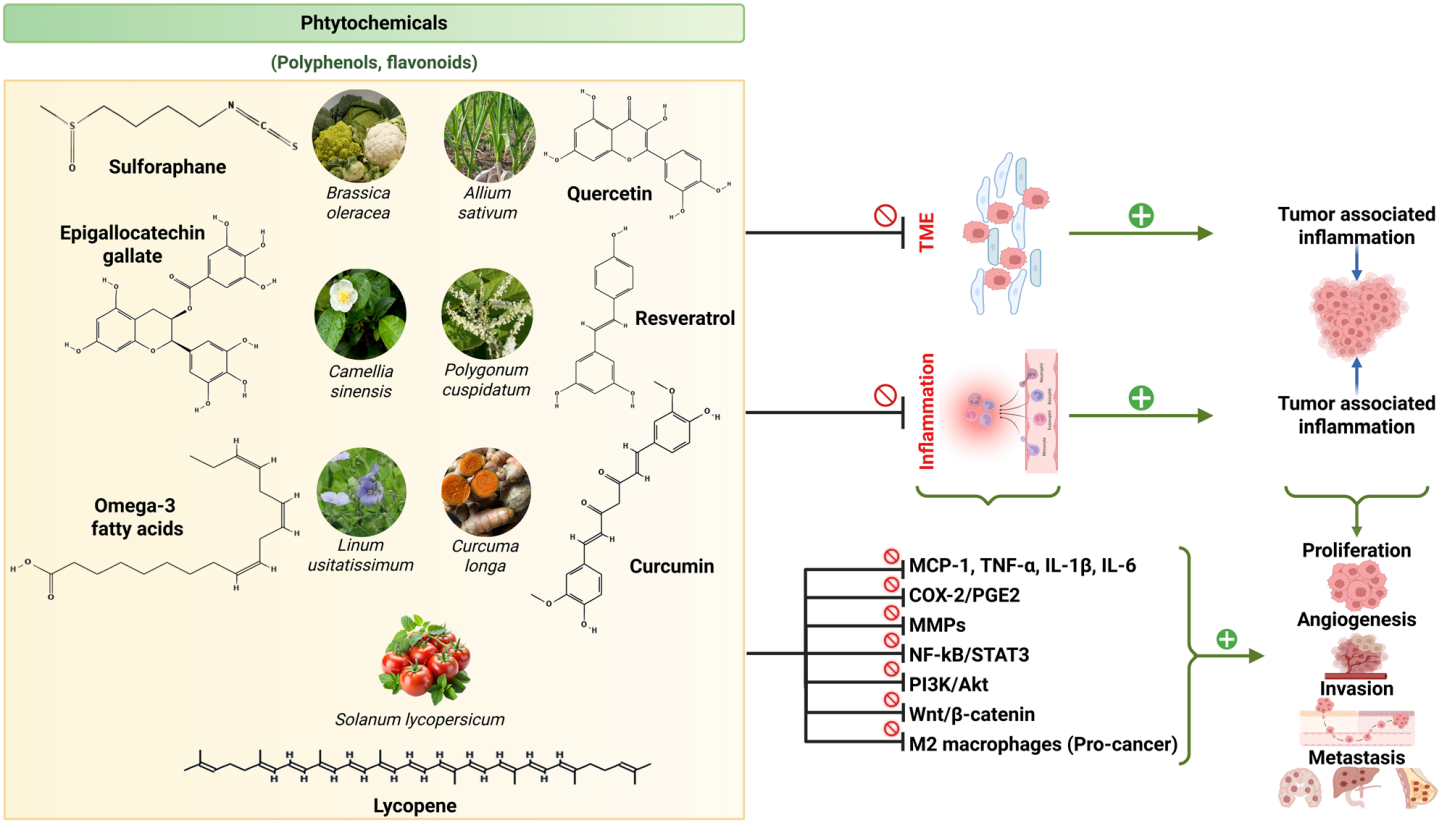
Simplified illustration highlighting the potential role of phytochemicals in inhibiting inflammation-induced tumors. Tumor-associated inflammation can be significantly reduced by specifically targeting inflammatory cytokines. Levels of MCP-1, TNF-α, IL-1β, IL-6, COX-2, and PGE_2_ can all be lowered by phytochemicals, including quercetin, resveratrol, curcumin, sulforaphane, epigallocatechin gallate (EGCG), and lycopene demonstrating their anti-inflammatory capabilities in the context of chronic inflammation. These effects lead to the regulation of the PI3K/Akt, NF-κB/STAT3, and Wnt/β-catenin signaling pathways by these phytochemicals, which could inhibit cancer initiation, proliferation, angiogenesis, invasion, and metastasis. Abbreviations: PGE_2_, prostaglandin E2; COX-2, cyclooxygenase-2; MMPs, matrix metalloproteinases; MCP-1, monocyte chemoattractant protein-1; STAT3, signal transducer and activator of transcription 3; TNF-α, receptor-associated factor-α; IL, interleukin; PI3K, phosphoinositide 3-kinase; Akt, protein kinase B; TME, tumor microenvironment

In contrast, plant-derived compounds significantly influence inflammation, with promising chemo-preventive potential [222]. Targeting inflammatory cytokines is an effective strategy for mitigating tumor-associated inflammation. Phytochemicals notably flavonoids, resveratrol, and curcumin have been revealed to suppress inflammatory cytokine production by modulating NF-κB and STAT3 signaling pathways [222]. Polyphenols, in particular, regulate signaling pathways including PI3K/Akt, NF-κB, MAPK, and Wnt/β-catenin, which are central to cancer development and progression [349] (Fig. 6).

Investigations have proven the efficacy of certain herbs, such as *Allium sativum* (garlic), *Curcuma longa* (turmeric), and *Apis mellifera* venom, in combating inflammation and associated disorders, including cancer [44]. A systematic review of human clinical trials carried out between 1980 and 2019 revealed that curcumin supplementation increased IL-10 levels and decreased MCP-1, TNF-α, IL-6, and CRP levels, reflecting its anti-inflammatory properties in chronic inflammation [350]. Curcumin not only reduces cancer risk but also sensitizes tumors to radiotherapy and chemotherapy. In fact, curcumin has been proposed as a complement to oxaliplatin- or 5-FU-based regimens in individuals who suffer advanced colorectal cancer, as demonstrated by favorable responses in a Phase I trial involving a four-month curcumin-based therapy [351].

**Fig. 7:**
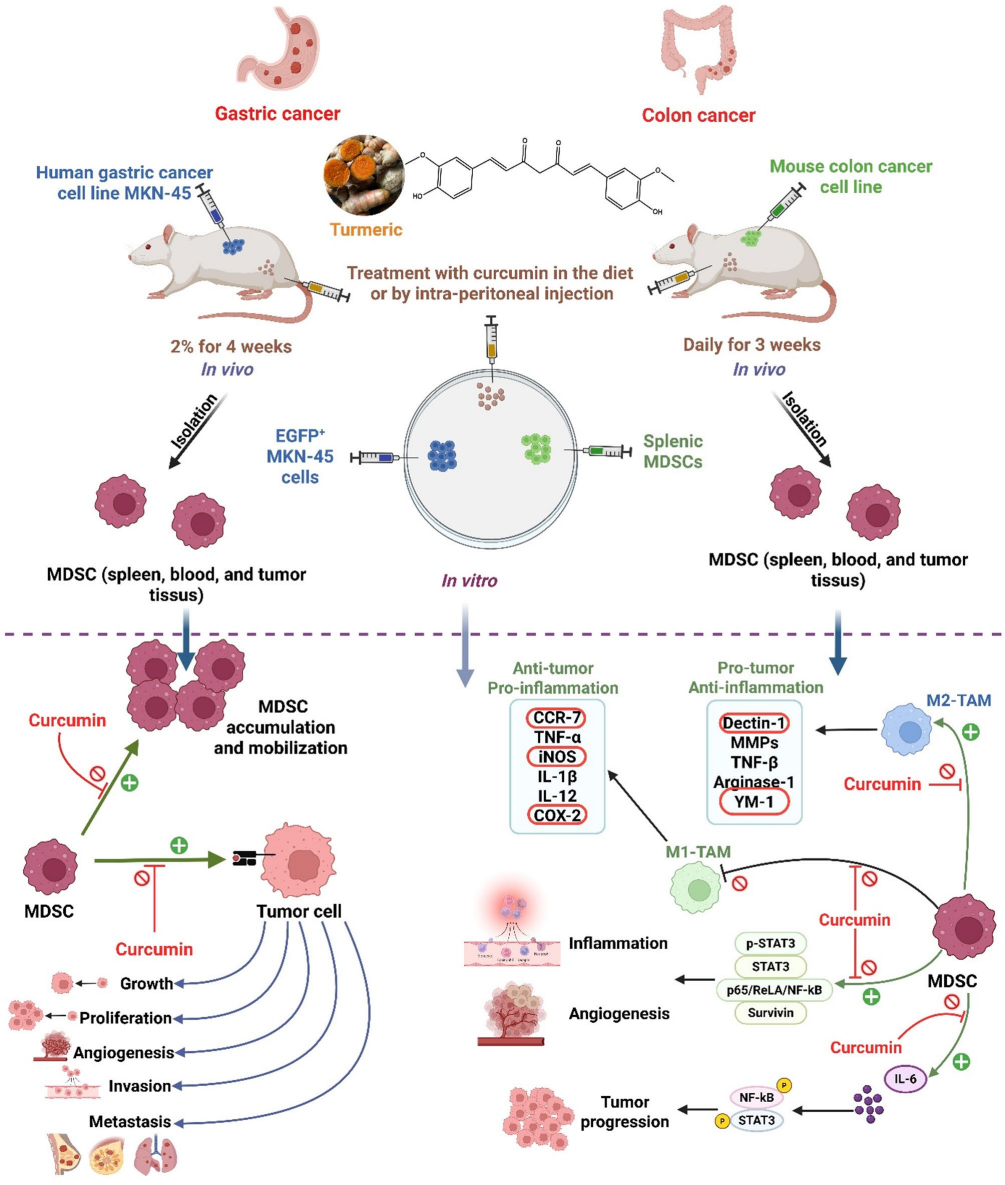
Graphical overview of the molecular mechanisms by which curcumin exerts its anti-cancer effect within MDSCs, using in vivo and in vitro assays. iNOS, COX-2, CCR7 and pro-inflammatory cytokines such as IL-1β and TNF-α are among the distinctive molecules expressed by M1 macrophages, whereas M2 macrophages express arginase-1, YM-1, TGF-β, MMPs and dectin-1. In MDSCs, curcumin downregulated YM-1 expression while upregulating iNOS and COX-2 expression. Therefore, one possible pathway for the anti-cancer activities of curcumin is the polarization of MDSCs into an M1 phenotype. The polarization of MDSCs towards an M1-like phenotype may have been caused, in part, by the inhibition of the Stat3 pathway by curcumin. Furthermore, NF-κB and STAT3 signaling pathways in MDSCs are inhibited by curcumin, which also reduces IL-6 production. This prevents tumor formation and the IL-6/Stat3 pathway in cancer cells. Curcumin reduces tumor growth, prevents cancer cells from interacting with MDSCs, induces MDSC differentiation, and suppresses MDSC activation. According to these findings, curcumin inhibits carcinogenesis by a novel pathway, and targeting MDSCs could be a useful approach for cancer treatment and prevention. Abbreviations: MDSCs, myeloid-derived suppressor cells; MMPs, matrix metalloproteinases; STAT3, signal transducer and activator of transcription 3; TNF-α, receptor-associated factor-α; IL, interleukin; iNOS, inducible nitric oxygen synthase; TAM, tumor-associated macrophage; M1, macrophage 1; M2, macrophage 2; TGF-β, transforming growth factor-β; NF-KB, nuclear factor-kappa B; COX-2, cyclooxygenase-2

In a current investigation using breast cancer cell lines (MCF-7 and MDA-MB-231), curcumin demonstrated anti-inflammatory and anti-cancer characteristics by inhibiting paclitaxel chemoresistance induced by M2-type TAMs. This effect was mediated *via* the knockdown of the PI3K-AKT/STAT3 signaling pathway [352]. Similarly, MDSCs aggregate within the spleen and tumor sites, promoting tumor development, angiogenesis, and progression [353]. The growth and invasion of MDSCs are regulated by key signaling pathways, including CXCR2, COX-2/PGE, VEGF, and JAK/STAT [354]. STAT3 serves as a central transcription factor governing the immunosuppressive activity of myeloid cells [355]. Various antagonists targeting the STAT3 pathway have been demonstrated to decrease the abundance of granulocytic-MDSCs [356]. Curcumin treatment not only suppressed MDSC proliferation but also inhibited STAT3 and NF-κB activation, along with IL-6 production. Both in vitro and in vivo investigations revealed that curcumin administration polarized MDSCs toward an M1-like phenotype, characterized by enhanced C-C chemokine receptor type 7 (CCR7) expression and reduced Dectin 1 expression [357] (Fig. 7).

In addition, resveratrol, a polyphenolic stilbenoid, partially inhibits T cells from releasing pro-inflammatory cytokines, making it a potential anti-inflammatory agent for preventing inflammation-related chronic disorders, including cancer. Its mechanism involves the inhibition of RelA acetylation *via* SIRT1 activation, which promotes the degradation of inhibitory protein-κBα (IkBα). This process reduces the expression of MMPs, COX-2, IL-1β, IL-6, and TNF-α through suppression of the NF-κB signaling pathway [358].

Besides, omega-3 fatty acids exhibit a primordial action in modulating inflammation by reducing eicosanoid synthesis. This reduction impacts pathways like AP-1 and NF-κB signaling, suppresses angiogenesis, and decreases the release of pro-inflammatory arachidonic acid metabolites including PGE_2_ and lipoxin B4 (LTB4) [359]. Furthermore, research highlights that omega-3 fatty acid supplementation significantly reduces colorectal adenoma incidence in individuals with low baseline plasma concentrations of these fatty acids [360].

Quercetin, a flavonoid widely distributed in vegetables, fruits, teas, and beans, has powerful anti-inflammatory qualities [361]. It suppresses liver inflammation by targeting the NF-κB/TLR/NLRP3 (NOD, LRR- and pyrin domain-containing protein 3) signaling pathway, reduces oxidative stress *via* the PI3K/Nrf2 pathway, downregulates mTOR signaling, and inhibits apoptotic markers implicated in liver malignancies [362]. In hepatocellular carcinoma models, quercetin eradicated cancer cells by activating the signaling pathway JAK/STAT, which regulates cell proliferation. Specifically, quercetin supplementation modulated tyrosine phosphorylation of STAT1 and enhanced IFN-β-mediated STAT1 activation in HepG2 cells, further promoting the signaling pathway JAK/STAT [363].

Moreover, sulforaphane has also demonstrated anticancer and anti-inflammatory properties in several types of cancer, such as colon, prostate, pancreatic, and bladder cancers. This dietary isothiocyanate derived from glucoraphanin is found in broccoli and other cruciferous vegetables [364, 365]. Sulforaphane can reduce the expression of pro-inflammatory cytokines, like the TNF-α and IL-6, by inhibiting NF-κB and TLR4/MyD88 pathways. Also, sulforaphane inhibits interferon regulatory factor 3 (IRF3) activation, downregulates COX-2 expression, and promotes the degradation of IL-1R-associated kinase-1 (IRAK-1), a crucial protein for inflammatory signaling. Moreover, sulforaphane acts as an anti-inflammatory molecule by inhibiting TLR4 oligomerization through the PI3K/Akt pathway and HIF-1α [365]. Sulforaphane also activates the transcription factor Nrf2. This key redox homeostasis regulator induces phase II detoxification enzymes such as quinone reductase and glutathione S transferase, which are also responsible for its anti-carcinogenic properties by enhancing the body’s antioxidant response and preventing inflammatory response [366].

Another polyphenol of the catechin family, the epigallocatechin gallate (EGCG), found in green tea plants (*Camellia sinensis*) and skins of some fruits and vegetables, demonstrates a crucial anti-inflammatory response through the inhibition of immune cell recruitment and activation, decreasing of pro-inflammatory cytokines releasing (TNF-α, IL-6, IL-8, IL-1β, MCP-1, and MMPs), and reducing reactive species generating (ROS and RNS) mainly regulated by modulation several pathways implicated in inflammation-induced cancer [364, 367]. Indeed, EGCG suppresses the activation of NF-κB, PI3K/Akt, and ERK1/2 pathways and blocks AP-1 activity, thereby reducing levels of pro-inflammatory enzymes such as iNOS and COX-2 and scavenges reactive species in colon and prostate cancer cells [367–369]. In addition, in melanoma models, EGCG eradicated cancer cells by targeting the JAK/STAT pathway to decrease PD-L1 expression in tumor cells, thereby enhancing T-cell activity. This inhibition occurs through a decrease in the expression and phosphorylation of STAT1, leading to reduced levels of IRF1, a key regulator of PD-L1 and PD-L2, in both human and murine melanoma cells. Moreover, in vivo experiments demonstrate that the antitumor effects of EGCG are dependent on CD8 + T-cells and are comparable to anti-PD-1 therapy [370].

Lycopene, a carotenoid found in red and pink vegetables and fruits like tomatoes, exhibits significant anti-inflammatory and antitumor activities [371]. Dietary intake of lycopene was associated with a lower prostate cancer risk [372]. In models of prostate cancer, lycopene treatment induces a decrease in the concentration of inflammatory mediators such as TNF-α, IL-1β, IL-6, IL-8, IL-10, and TGF-β associated with a reduction in tumor growth and an increase in survival [373]. Moreover, in SW480 human colorectal cancer cells, lycopene induces a decrease in the mRNA expression of iNOS and COX-2, resulting in a reduction in PGE_2_ and NO production [374]. Indeed, lycopene suppresses cancer cells by inhibiting the NF-κB and MAPK (ERK and JNK) signaling pathways, which regulate cell proliferation, differentiation, and survival and play crucial roles in the inflammatory response [374]. These pathways activate various downstream genes that contribute to the production of inflammatory mediators and immune cell activation, ultimately influencing the progression of inflammation and the adaptive immune response. Indeed, lycopene induces an increase in the accumulation of immune cells, specifically NK cells, macrophages, and neutrophils, leading to reduced tumor growth and improved survival in tumor-bearing mice [373].

Additionally, many other compounds have demonstrated anti-inflammatory and anti-tumorigenic effects, such as β-carotene, gingerol, capsaicin, berberine, and garlic, among others, by targeting key signaling pathways, including NF-κB, MAPK, and JAK/STAT, which inhibit anti-inflammatory mediators and contribute to their anticancer activity [375, 376].

These bioactive compounds, whether derived from dietary or pharmaceutical sources, consist of complex chemical structures. These structures often yield varied and sometimes opposing effects on cell growth and death, frequently mediated by their influence on the expression of numerous genes through direct or indirect mechanisms.

**Table 1:** Key Anti-Inflammatory phytochemicals in Cancer therapy

Phytochemical	Source/Origin	Mechanism of Action	Cancer Types Studied	Key Findings	References
Curcumin	Turmeric (*Curcuma longa*)	- Modulated NF-κB, STAT3, and PI3K/Akt pathways - Increased IL-10 - Decreased MCP-1, TNF-α, IL-6, CRP levels	Breast, colorectal	- Enhanced chemoradiotherapy sensitivity - Reduced inflammation and chemoresistance - Polarized MDSCs to M1 phenotype; tested in Phase I trials for efficacy	[351] ; [357] ; [350] ; [352]
Resveratrol	Grapes, berries, peanuts	- Inhibited RelA acetylation *via* SIRT1 activation - Suppressed NF-κB signaling - Reduced MMPs, COX-2, IL-1β, IL-6, TNF-α	Chronic inflammation-related cancers	- Prevented T-cell-mediated pro-inflammatory cytokine release - Decreased inflammation and tumor proliferation	[358]
Omega-3 Fatty Acids	Fish oil, algae oil	- Reduced eicosanoid synthesis - Modulated NF-κB, AP-1 pathways - Suppressed angiogenesis and arachidonic acid metabolites	Colorectal	- Significantly lowered colorectal adenoma incidence in individuals with low baseline omega-3 levels	[359] ; [360]
Quercetin	Fruits, teas, beans, vegetables	- Targeted NF-κB/TLR/NLRP3 and PI3K/Nrf2 pathways - Modulated STAT1 activation - Inhibited mTOR signaling	Liver (Hepatocellular carcinoma)	- Reduced liver inflammation and oxidative stress - Enhanced IFN-β-induced STAT1 activation - Promoted JAK/STAT signaling for cancer cell eradication	[363] ; [362] ; [361]
Sulforaphane	Cruciferous vegetables (broccoli, brussels sprouts, kale, and cauliflower)	- Inhibited NF-κB/TLR4/MyD88 pathway, and promoted IRAK-1 degradation - Inhibited TLR4 oligomerization *via* PI3K/Akt and HIF-1α - Reduces TNF-α, IL-6, and COX-2 Activated Nrf2 Inhibited IRF3 activation	Colon Prostate Pancreatic Bladder	- Suppressed inflammation and oxidative stress - Boosted immune response by enhancing phagocytosis in macrophages and NK cells in leukemia mouse models - Reduced cancer cell migration and invasion and tumor growth Promoted apoptosis in cancer cells	[364]; [365]; [366]; [377]; [378];
Epigallocatechin gallate (EGCG)	Green tea (*Camellia sinensis*) and skins of onions, apples, plums	- Inhibited NF-κB, AP-1, PI3K/Akt, ERK1/2, and JAK-STAT1/IRF1 pathways - Suppressed TNF-α, IL-6, IL-8, IL-1β, MCP-1, and MMPs - Decreased iNOS, COX-2, and PGE_2_ - Reduced ROS/RNS production - Inhibited recruitment and activation of immune cells Decreased PD-L1/PD-L2 expression in melanoma	Colon Prostate Melanoma	- Demonstrated potent anti-inflammatory and antioxidant activities - Enhanced CD8 + T-cell response; comparable efficacy to anti-PD-1 therapy in vivo - Inhibited tumor growth and inflammation; modulated angiogenesis and metastasis	[364]; [367]; [369]; [370]
Lycopene	Red/pink fruits and vegetables (tomatoes)	- Reduced TNF-α, IL-1β, IL-6, IL-8, IL-10, and TGF-βInhibited iNOS, COX-2, PGE_2_, and NO production - Suppressed NF-κB and MAPK (ERK and JNK) pathways - Enhanced NK cells, macrophages, and neutrophil infiltration	Prostate, Colorectal	- Demonstrated anti-inflammatory, immune-modulating, and tumor-suppressing effects Reduced tumor growth and improved survival in murine models	[371]; [373]; [374]

## Challenges and prospects in Cancer therapy targeting inflammation

The human body exhibits diverse and dynamic responses when medications are introduced, reflecting its complex biological characteristics. This complexity is further compounded when modeling animals with varying inflammatory phenotypes. These challenges necessitate careful consideration of factors such as dosing adjustments, outcome measurements, and evaluation techniques. Recent studies have focused on simulating a quantitative and simplified process of anti-inflammatory therapy, emphasizing the importance of monitoring changes across different components of tumor cells. Such models show promise for incorporating a broader range of phenotypes, leading to deeper insights [379].

The efficacy of these therapies has been evaluated using non-invasive methods, including imaging, circulating inflammatory markers, and varied pain scores. This approach has been optimized for efficient animal research by analyzing inflammation across different levels and time points, particularly within virtual populations. Furthermore, animal models derived from human physiology can serve as effective tools for validating the efficacy of medications targeting inflammation, as they replicate the intricate pathophysiology observed in humans. However, these models face inherent limitations, including their reduced capacity to fully mimic human physiology, especially in complex diseases like cancer and inflammation [380].

To address these challenges, advanced cancer models have been developed that integrate mathematical frameworks and biological processes. These models account for cellular heterogeneity by describing phenomena in one, two, or three dimensions. They also consider the signaling relay nodes involved within and between cells, including the ECM, immune cells, and stromal cells. Importantly, these models incorporate mechanisms such as drug diffusion, cellular drug uptake, and extracellular drug metabolism to better simulate the TME.

Cancer stem cells are considered to have a key function in the initiation, progression, and metastasis of various cancers. Tumor heterogeneity, driven by this cellular diversity, represents a major factor in therapeutic resistance. Evidence has confirmed the existence of intercellular communication within tumor spheroids or organoids, making it challenging to achieve consistent therapeutic outcomes [381]. Therefore, successful inflammation-targeted cancer therapies require a dual approach that combines anti-inflammatory drugs with antitumor agents. However, ensuring the anti-cancer efficacy of these therapies necessitates rigorous assessments of tumor responses, including evaluations of toxicity and therapeutic effectiveness.

## Addressing resistance mechanisms

Inflammation holds significant potential as a therapeutic strategy to combat cancer. However, determining the most effective approach to harness inflammation for tumor control while minimizing damage to healthy cells remains a challenge. Targeting the inflamed TME could provide a highly effective therapeutic approach, allowing the rest of the body to maintain lower inflammation levels [379]. Achieving success in this area necessitates innovative clinical trial designs to evaluate the effectiveness of monotherapy versus combination therapies across various cancer types.

The immunological microenvironment of the tumor is currently a pivotal aspect of cancer research, given the tendency of tumor cells to manipulate nearby normal cells to satisfy their survival and proliferation requirements. Targeting the inflamed TME offers an optimal therapeutic ratio by reducing systemic inflammation. Existing anti-inflammatory therapies, such as those employed in rheumatology, could be rapidly repurposed for oncological applications. For example, pathways like TLR signaling and COX signaling—along with agents such as indomethacin and aspirin—have demonstrated cancer-preventive effects [25, 153].

Emerging technologies, genomic integration, and publicly available datasets further enhance cancer’s candidacy for targeted therapeutic intervention. The well-documented correlation between inflammation and cancer underscores the potential of inflammation as a therapeutic target. While leukocyte mono-treatment alone has demonstrated limited efficacy, its combination with adjunctive therapies such as endostatin or radiation therapy significantly improves outcomes. Although monotherapy can yield positive results in certain patients, a notable trial illustrated that combining it with chemo-radiotherapy yielded superior effectiveness [200].

Monotherapy, however, is not without risks, as it can induce tumor resistance by reprogramming leukocytes from a Th1 (pro-inflammatory) to a Th2 (anti-inflammatory) response pattern. Furthermore, safety concerns about these treatments persist. Suppressing anti-inflammatory responses can inadvertently reduce the likelihood of achieving effective cancer therapy. Targeting macrophages has emerged as a promising cancer treatment strategy. Tamoxifen, for instance, is particularly advantageous because of its cross-brain barrier capability, making it effective against brain tumors and limiting cancer cell infiltration into the brain.

While initial anti-cancer therapies can demonstrate substantial efficacy, resistance mechanisms often emerge, leading to treatment failure [382]. Resistance to inflammation-based anti-cancer therapies may involve feedback loops, mutations, and other mechanisms. Current research focuses on overcoming these challenges through logical drug combinations, targeting specific nodes in the tumorigenic network, and employing “adaptive therapy,” an approach that modulates treatments in response to evolving resistance [383–386].

## Concluding remarks and perspectives

Targeting inflammation constitutes a compelling option for cancer prevention and management, as most cell types implicated in cancer-related inflammation remain genetically unaltered and develop reduced susceptibility to drug resistance. Inflammatory responses, depending on their context, can either enhance or inhibit tumor progression. The balance between pro-tumorigenic and anti-tumorigenic actions is largely governed by the potency of inflammatory mediators and the nature and intensity of molecules regulating the inflammatory response. Acute inflammation, a normal physiological reaction to harmful stimuli, may exert anti-tumorigenic effects in the first phases of cancer development by eliminating precancerous cells. Conversely, within typical chronic inflammatory conditions, the presence of inflammatory cells often supports tumor survival, proliferation, and metastasis. Future research will undoubtedly focus on elucidating the molecular and cellular mechanisms that modulate inflammation and the activity of immune and inflammatory cells, particularly concerning early tumorigenesis, metastatic dissemination, and metastatic expansion. These investigations hold promise for identifying novel biomarkers and therapeutic targets to increase the efficiency of cancer therapies. However, progress in this area faces significant challenges, including the lack of robust in vivo and clinical studies, the technical difficulties associated with single-cell analysis in complex tissues, and delays in studying native metastases. Overcoming these limitations will be critical for a deeper insight into how inflammatory and immunological pathways contribute to treatment resistance, especially as the spectrum of anti-cancer treatments is constantly expanding, incorporating immune-based interventions.

This review has outlined key principles governing the interplay between inflammation and cancer, highlighting how inflammation can paradoxically support both immune surveillance and tumor development. We have established links between inflammation’s “normal” physiological roles—such as maintaining immunity and tissue homeostasis—and its pathological contributions to cancer initiation and progression. Moving forward, efforts should aim to distill these complex and evolving insights into a coherent framework, identifying core concepts that regulate inflammatory processes and the molecular and cellular mechanisms driving tumorigenesis. This approach will pave the way for pioneering developments in therapeutic regimens that exploit inflammation’s dual role, ultimately enhancing our ability to combat cancer effectively.

A promising pathway beyond suppression is the active resolution of inflammation, particularly through SPMs. In addition, more precise control of inflammatory pathways in the tumor microenvironment is provided by biologics and targeted drugs such as IL-6 or JAK inhibitors. Future studies should focus on the development of combinatorial drugs that rebalance immune signaling in favor of tumor suppression and on the use of omics technology to identify unresolved inflammation.

## Data Availability

All data were used and cited in the manuscript.

## Abbreviations

ABCA1: ATP-Binding Cassette Transporter A1
ABCG1: ATP-Binding Cassette Transporter G1
Acml: Atypical chronic myeloid leukemia
AIPs: Anti-inflammatory peptides
Akt: Protein kinase B
AML: Acute myeloid leukemia
ANN: Artificial neural networks
ATRA: All-trans-retinoic acid
Bcl-2: B-cell lymphoma-2
Bcl-xL: B-cell lymphoma-extra large
BCR: B cell receptor
CAD: Computer-aided diagnosis
CCD: Coiled-Coil Domain
CID: Chronic inflammatory disease
CLL: Chronic lymphocytic leukaemia
CNL: Chronic neutrophilic leukemia
CNN: Convolutional neural network
COX: Cyclooxygenase
CRP: C-reactive protein
CXCL: C-X-C motif chemokine ligand
CXCR: C-X-C motif chemokine receptor
DAMPs: Damage-associated molecular patterns
DCs: Dendritic cells
DHA: Docosahexaenoic acid
DL: Deep learning
DLRI: Deep learning radiomic signature of inflammation
E2F3: E2F transcription factor 3
EGCG: Epigallocatechin gallate
EPA: Eicosapentaenoic acid
ERK: Extracellular signal-regulated kinase
ERK: Extracellular signal-related kinase
ESCC: Esophageal squamous cell carcinoma
FPRL1: Formyl peptide receptor-like 1
GPCRs: Specific G protein-coupled receptors
HIF-α: Hypoxia-inducible factorα
HMGCR: HMG-CoA reductase
HNSCC: Head and neck squamous cell carcinomas
IFN: Interferon
IKK: Inhibitory-κB kinase
IL: Interleukin
IRAK-1: IL-1R-associated kinase-1
IRF: Interferon regulatory factor
I-κBs: Inhibitors of kappa B
JAK: Janus Kinase
LXA4: Lipoxin A4
mAb: Monoclonal antibody
MAPKs: Mitogen-activated protein kinases
MCP-1: Monocyte chemoattractant protein-1
MDSCs: Myeloid-derived suppressor cells
ML: Machine learning
MMPs: Metalloproteinases
mTOR: Mammalian target of rapamycin
NF-κB: Nuclear factor kappa B
NK: Natural killer
NOS: Nitrous oxide synthase
NSAIDs: Non-steroidal anti-inflammatory drugs
NSCLC: Non-small cell lung cancer
p38: Protein 38
p65: Protein 65
PAMPs: Pathogen-associated molecular patterns
PCNA: Proliferating cell nuclear antigen
PDAC: Pancreatic ductal adenocarcinoma
PFS: Progression-free survival
PGE_2_: Prostaglandin E2
PI3K: Phosphoinositide 3-kinase
PRRs: Pattern recognition receptors
PTC: Papillary thyroid cancer
PTEN: Phosphatase and tensin homolog deleted on chromosome ten
PUFAs: Polyunsaturated fatty acids
RHD: Rel homology domain
ROS: Reactive oxygen species
RTK: Receptor tyrosine kinases
RvD1: Resolvin D1
SH2: Src 2 homology domain
SIRT1: Sirtuin 1
SPMs: Specialized pro-resolving mediators
SR-BI: Scavenger Receptor Class B Type I
STAT3: Signal transducer and activator of transcription 3
TAD: Transactivation domain
TAMs: Tumor-associated macrophages
TAPI-1: TNF-α processing inhibitor-1
TCM: Traditional Chinese medicine
Tfh: T follicular helper
TGCTs: Testicular germ cell tumors
TGF-β: Transforming growth factor-β
Th: T helper
TILs: Tumor-infiltrating lymphocytes
TLR: Toll-like receptor
TME: Tumor microenvironment
TNBC: Triple-negative breast cancer
TNFR: Tumor necrosis receptor
TNF-α: Tumor necrosis factor-α
Tregs: Regulatory T cells
VEGF: Vascular endothelial growth factor
